# Silencer variants are key drivers of gene up-regulation in Alzheimer’s disease

**DOI:** 10.1126/sciadv.adz3323

**Published:** 2026-02-11

**Authors:** Di Huang, Ivan Ovcharenko

**Affiliations:** Division of Intramural Research, National Library of Medicine, National Institutes of Health, Bethesda, MD 20892, USA.

## Abstract

The genetic mechanisms of ~90% of Alzheimer’s disease (AD)–associated variants residing in noncoding DNA remain poorly understood. To address this, we developed a deep learning framework that integrates bulk histone modification data with single-cell open chromatin profiles to evaluate the regulatory potential of noncoding variants. This model identified 1457 silencer and 3084 enhancer AD-associated variants in dorsolateral prefrontal cortex, classifying gene loci as silencer-only (SL), enhancer-only (EN), or dual-function (ENSL). EN loci predominantly regulate housekeeping metabolic processes, SL loci (including *MS4A6A* and *HLA-D*) are linked to immune responses (with ~70% substantially up-regulated in AD microglia), while ENSL loci are implicated in neurofibrillary tangle assembly. Our model achieves robust power in assessing the impact of regulatory variants, with ~70% directional concordance with experimental results. It identified rs636317 as a putative causal silencer variant, distinguishing it from a neutral variant located 11 base pairs away. This study advances understanding of the AD-associated regulatory landscape and provides a framework for ascertaining noncoding variants in AD pathogenesis.

## Teaser

AI model of causal gene regulatory variants highlights the key role of silencers in Alzheimer’s disease.

## INTRODUCTION

Alzheimer’s disease (AD) is the most common neurodegenerative disorder among the elderly, representing a rapidly escalating global epidemic (*1*–*3*). It is projected that, by 2060, 13.8 million Americans will be affected by AD, imposing substantial societal and economic burdens (*4*). Pathologically, AD is characterized by two hallmark features—the accumulation of amyloid-β (Aβ) into extracellular plaques and the aggregation of hyperphosphorylated tau into neurofibrillary tangles within neurons (*5*). Beyond these features, AD brains exhibit profound dysregulation of immune responses, impaired glucose and lipid metabolism, and vulnerable brain vasculature, among other abnormalities (*6*–*9*). The etiology of AD is profoundly complex and multifactorial, posing great research challenges (*8*, *10*).

Because of the high genetic heritability of AD (58 to 79%, varying across investigation contexts) (*11*), genetic analyses have emerged as a powerful tool for unraveling its underlying mechanisms (*12*). These approaches have identified pivotal AD-associated genes and biological pathways. However, the precise molecular underpinnings of AD remain incompletely understood. In particular, most AD-associated variants reside in intronic or intergenic regions, and their regulatory roles remain largely unexplored (*13*). To bridge this knowledge gap, multiomic approaches, such as genome-wide transcriptomic and epigenomic data, have been leveraged to elucidate how these noncoding variants influence gene expression in AD brains (*14*–*16*). Recent advances in single-cell/nucleus sequencing techniques (scRNA-seq/snRNA-seq and scATAC-seq) have provided critical insights into cell type–specific contributions to AD pathological burdens (*13*, *17*–*19*). For example, transcriptomic analysis for cerebrovascular cells has linked brain-blood barrier breakdown with *APOE4*-dependent cognitive decline (*17*). Neurons, especially excitatory neurons, experience substantial losses in chromatin accessibility in AD brains (*13*). Despite these advances, existing single-cell epigenomic studies largely focus on chromatin accessibility, leaving the directionality (activating versus repressing) of regulatory effects unexplored.

Furthermore, massively parallel reporter assays (MPRAs) have been used to simultaneously assess the regulatory influence of up to tens of thousands of noncoding variants. While these assays have identified variants with substantial effects on gene expression (*20*–*23*), most MPRA experiments have been conducted in immortalized cell lines (e.g., HEK293, K562, GM12878, HepG2, SK-N-SH, etc.), raising concerns about the relevance of MPRA findings to in vivo conditions. A few studies have extended the MPRA technique to primary tissues or organoids, such as fetal brains (*24*, *25*). The MPRAbase collection of MPRA experiments across multiple cell lines (including human or mouse neuronal and brain cells) (*24*) provides an important resource for further comparative analysis of potential limitations stemming from an immortalized cell line analysis.

Here, to assess the functional roles of intronic and intergenic variants in the dorsolateral prefrontal cortex (DLPFC), we developed a deep learning model that integrates large-scale complementary epigenomic profiles at bulk and single-cell levels. This approach identifies thousands of enhancer and silencer variants among over 18,000 single-nucleotide variants (SNVs) associated with AD in genome-wide association studies (GWASs). The distribution of these variants categorizes AD susceptibility loci into three distinct classes. Each class, associated with unique transcriptomic and epigenomic patterns in the healthy and AD DLPFCs, exhibits specific molecular and cellular functions during AD progression. By prioritizing candidate causal regulatory variants for AD, this study sheds light on the regulatory mechanisms underlying AD pathogenesis.

## RESULTS

### Deep learning profiles AD-associated regulatory variants in the DLPFC

**Fig. 1.**
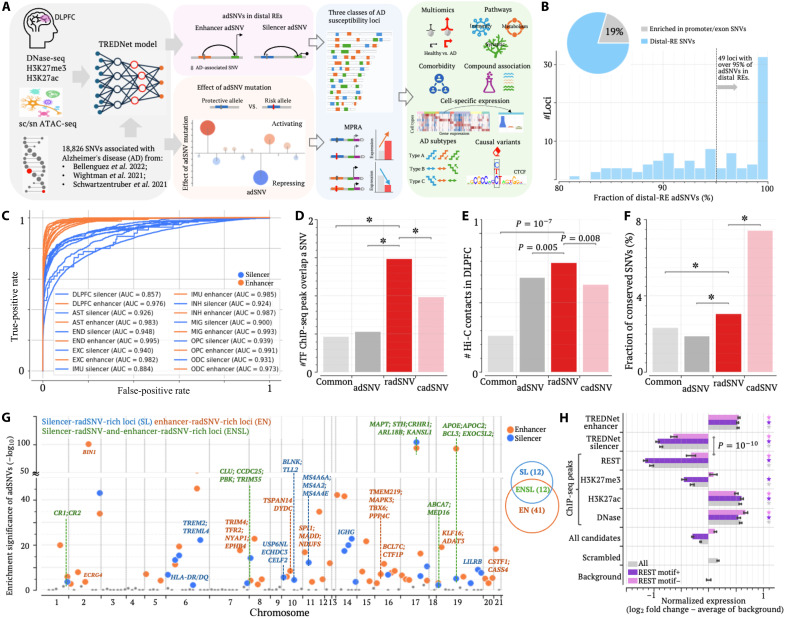
Profiling silencer and enhancer adSNVs with TREDNet. (**A**) Schematics of the analysis workflow for the identification of radSNVs in the DLPFC and subsequent analyses. DNase-seq, DNase I hypersensitive sites sequencing. (**B**) Distribution of adSNVs in distal REs and exon/promoter regions within individual AD susceptibility loci. (**C**) Classification performance (auROCs) of the DLPFC TREDNet for silencers and enhancers in the DLPFC and its cell types. auPRCs are in fig. S1 and table S2. AUC, area under the curve; AST, astrocytes; END, endothelia; EXC, excitatory neurons; IMU, immune cells; INH, inhibitory neurons; MIC, microglia; OPC, oligodendrocyte progenitors; ODC, oligodendrocytes. (**D**) Overlap of TF ChIP-seq peaks per SNV across SNV groups. (**E**) Chromatin contact frequencies across SNV groups. (**F**) Proportion of SNVs located within evolutionarily conserved regions. In (E) and (F), adSNV and common represent adSNVs and common SNVs located within neither exon nor promoter regions. In (D) to (F), *P* values (*P < 10^–10^) were determined against common SNVs using two-sided binomial tests without adjustment. (**G**) Genomic distribution plot for AD susceptibility loci. Different locus classes are represented by different colors. (**H**) MPRA activity scores for different element groups. Data are presented with the median ± SEM. *P* values (*P < 10^–10^) were determined against background using the two-sided Student’s *t* test without adjustment (*n* = 756).

For a comprehensive investigation, we compiled AD-associated variants from three recent GWASs, each involving hundreds of thousands to millions of participants (*15*, *16*, *26*). In total, we collected 18,826 SNVs significantly associated with AD in at least one of these studies and referred to them as adSNVs (Fig. 1A). Consistent with prior observations, 91.1% of adSNVs reside in intronic or intergenic regions, categorized as distal–regulatory element (distal-RE) variants.

To further examine the distribution of adSNVs, we defined a gene locus for each gene annotated in the GENCODE project (*27*) as the gene body plus its upstream and downstream flanking regions. After merging adjacent gene loci containing adSNVs, we defined 124 distinct AD susceptibility loci (see Materials and Methods and table S1). On average, each AD susceptibility locus spans 636 kb and 6.4 gene loci, with lengths ranging from 29.6 kb to 3.3 Mb. Among these AD susceptibility loci, 24 (19%) are enriched for promoter/exon adSNVs, while 49 loci (40%) predominantly harbor distal-RE variants (see Materials and Methods), with more than 95% of adSNVs located in distal-REs (Fig. 1B). These observations underscore the prominent roles of distal-RE adSNVs in AD pathogenesis, emphasizing the imperative to decipher their regulatory functions.

To annotate distal-RE adSNVs, we adapted a two-phase deep learning model TREDNet (*28*) to predict enhancers and silencers in the human DLPFC and its major cell types, including excitatory and inhibitory neurons, astrocytes, endothelial cells, microglia, oligodendrocytes, oligodendrocyte progenitor cells, and immune cells. For training, we compiled deoxyribonuclease (DNase) I hypersensitive site sequencing peaks (DNase-seq) and chromatin immunoprecipitation sequencing (ChIP-seq) peaks for histone modifications H3K27ac (Histone H3 lysine 27 acetylation) and H3K27me3 (Histone H3 lysine 27 trimethylation) from postmortem brain samples of 20 elderly undemented cases (the average age at death was 89 years) as provided in the Religious Orders Study and Rush Memory and Aging Project (ROSMAP) (*3*). Two main objectives of this study are to build a baseline deep learning model for accurately predicting regulatory activity from genomic sequences and to quantify activity changes induced by disease-associated variants. Demented samples generally carry AD-associated variants and thus harbor altered H3K27ac and H3K27me landscapes. AD risk alleles are often absent from the human reference genome and thus cannot be accurately modeled in a human reference sequence–based analysis. Therefore, to minimize confounding effects from AD epigenomics, we excluded these data in the training whenever possible. scATAC-seq or snATAC-seq data for brain tissues from three recent studies (*18*, *29*, *30*) were also incorporated in the TREDNet.

Using a multitask cost function, the TREDNet model was optimized to predict enhancers and silencers in the DLPFC and its cell types (see Materials and Methods). The resulting model exhibited robust performance in cross-validation, achieving an area under the receiver operating characteristic curve (auROC) of 0.985 for enhancers and 0.899 for silencers and an area under the precision-recall curve (auPRC) of 0.885 for enhancers and 0.637 for silencers on average, under a positive-to-control sample ratio of 1:9, among test samples (Fig. 1C, fig. S1, and table S2). Of note, auROC and auPRC levels are positively correlated with GC (G and C nucleotides) content and negatively with repeat density of input DNA sequences (fig. S2), consistent with our report in other 97 human biosamples (*31*). In general, silencer sequences have a lower GC content level and higher repeat density than enhancers (fig. S2), which may partially explain the better prediction performance on enhancers than on silencers. In the next section, the performance of this DLFPC TREDNet will be further examined.

Applying the DLPFC TREDNet model to distal-RE adSNVs, we identified 1457 putative silencer and 3084 putative enhancer adSNVs in the DLPFC (table S3). These putative regulatory adSNVs (dubbed radSNVs) formed the primary focus of this study. As a reference, we assessed the functional importance of distal-RE adSNVs using the combined annotation-dependent depletion (CADD) scores (*32*), defining those with CADD > 5 as cadSNVs. This threshold was chosen to yield the number of cadSNVs comparable to that of radSNVs. On average, a radSNV overlaps with 1.5 transcription factor (TF) ChIP-seq peaks detected for the neuronal cell line SK-N-SH from the Encyclopedia of DNA Elements (ENCODE) project (*33*), representing a threefold enrichment over distal-RE adSNVs and common SNVs archived in the dbSNP database (*34*) (binomial test *P* < 10^–10^; Fig. 1D). Furthermore, among all examined variants (including cadSNVs), radSNVs exhibit the highest density of chromatin contacts detected in DLPFC cells (*35*) (binomial test *P* < 10^–10^; Fig. 1E) and the strongest enrichment in snATAC-seq peaks detected in the middle temporal gyrus (*36*) or across multiple brain regions (*37*) (both independent of the DLPFC TREDNet training; *P* < 10^–10^; fig. S3), as well as in brain DNA-demethylated regions (*P* < 10^–10^; fig. S3) (*38*).

Moreover, 3.1% of radSNVs reside in genomic regions conserved across 100 vertebrate species (*39*), significantly exceeding 1.9% of distal-RE adSNVs and 2.3% of distal-RE common SNVs (*P* ≤ 0.005; Fig. 1F), second only to 7.8% of cadSNVs (*P* < 10^–10^), whose extreme conservation reflects the prominent weighting of evolutionary constraint in CADD scores (*35*). Notably, 31.5% of radSNVs overlap snATAC-seq peaks conserved between human and mouse brains (*37*), exceeding distal-RE common SNVs (21.1%) and adSNVs (28.4%, *P* < 10^–10^) and slightly surpassing cadSNVs (30.4%, *P* = 0.43; fig. S3). These strong sequence and epigenomic conservations point to the functional importance of their host regulatory elements. radSNVs also display stronger GWAS AD association than other adSNVs (*P* ≤ 0.006; fig. S4). In addition, we analyzed the enrichment of predicted radSNVs in ChIP-seq peaks derived from H1-differentiated neural cells (the ENCODE project, sample ID: CL:0002319) (*33*) across 11 histone marks. Enhancer radSNVs are densely populated in the peaks of activating marks, including but not limited to H3K27ac (*P* < 0.0001 versus all adSNVs and cadSNVs; fig. S4). In contrast, silencer radSNVs are significantly enriched in the peaks of H3K27me3 (as expected) and H4K20me1, another repression-associated mark (*40*, *41*), supporting the validity of our radSNV predictions. Together, these results underscore the regulatory and phenotypic importance of radSNVs in brain cells.

Further analysis revealed distinct distribution patterns of silencer and enhancer radSNVs in AD susceptibility loci. We herein identified 12 loci enriched exclusively with silencer radSNVs (referred to as SL loci), 41 loci predominantly with enhancer radSNVs (EN loci), and 12 loci with both enhancer and silencer radSNVs (ENSL loci; Fig. 1G and table S1). Of note, an AD susceptibility locus often spans multiple adjacent AD-associated gene loci. ENSL loci, featuring an interplay between activating and repressive regulatory elements, encompass 255 AD-associated gene loci, including *ABCA7*, *APOE*, *APOC2*, *BCL3*, *CLU*, *CR1*, *CRHR1*, *MAPT*, *PTK2B*, etc. (*42*). SL loci comprise 115 gene loci in total, including the genes that have been implicated in neuroinflammation and neuroimmune dysregulation in AD brains, such as *HLA-D*, *MS4A6A*, *TREM2*, *TREM4L*, and *USP6NL* (*42*). EN loci include 188 gene loci, featuring prominent AD-associated genes like *BIN1*, *BCL7*, and *CASS4*.

### Evaluating the performance of DLPFC TREDNet with MPRA results

Before further examining biological contributions of radSNVs and AD susceptibility loci, we benchmarked the DLPFC TREDNet model using MPRAduo data, which use enhancer-embedded reporter vectors to quantify regulatory activities (by the expression levels of reporter genes) for more than 12,000 RE1-Silencing Transcription Factor (REST) ChIP-seq peaks across four human cell lines, including the neural cell line SK-N-SH (*43*). MPRAduo activities below the background (i.e., the activities of random sequences) indicate regulatory repression, as exemplified by REST ChIP-seq peaks harboring canonical REST binding motifs (referred to as RESTmotif+ REST peaks), a well-established silencer class (*43*). By contrast, RESTmotif− DLPFC H3K27me3 peaks [retrieved from the ROSMAP project (*3*)] show insignificant repressive activities (Student’s *t* test *P* = 0.06), which is consistent with the published reports across cellular contexts (*41*, *43*–*45*) . These observations underscore the insufficiency of H3K27me3 alone to define silencers and highlight the ongoing challenge of silencer identification.

Unexpectedly, RESTmotif+ DLPFC H3K27ac peaks act as enhancers, surpassing the activation of the other H3K27ac peaks (Student’s *t* test *P* = 2 × 10^–6^; Fig. 1H), illustrating REST’s context-dependent roles, despite its motifs being depleted in DLPFC H3K27ac peaks in comparison to H3K27me3 peaks (binomial test *P* < 10^–10^). RESTmotif+ REST peaks are rare, limiting their utility in comprehensively mapping silencer landscapes. For example, we identified a total of 3665 RESTmotif+ REST ChIPs in the SK-N-SH cell line using the REST ChIP-seq data from the ENCODE project (*33*) and the HOCOMOCO v13 REST binding motif (see Materials and Methods) (*46*), covering merely two distal-RE adSNVs. These limitations collectively underscore the challenges of silencer identification and motivate the development of the DLPFC TREDNet model while also explaining its comparatively stronger performance of the DLPFC TREDNet in enhancer prediction than in silencer prediction*.*

MPRAduo sequences predicted as silencers by the DLPFC TREDNet (referred to as TREDNet silencers) show markedly reduced MPRAduo activities across all MPRAduo vector designs (Student’s *t* test *P* < 10^–6^ versus background; Fig. 1H and fig. S5) in SK-N-SH cells, second only to RESTmotif+ REST peaks. Notably, TREDNet silencers lacking REST motifs retain significant repression (Student’s *t* test *P* = 10^–47^ versus background), even in comparison to RESTmotif− REST peaks (*P* = 10^–10^; Fig. 1H), indicating that this model, trained by contrasting DLPFC H3K27me3 sequences against background and DLPFC H3K27ac sequences, captures the repressive sequence determinants beyond REST canonical motifs.

In GM12878, HepG2, and K562 cell lines, similar trends were observed: Neither H3K27me3 nor REST binding motifs reliably identify silencers, while TREDNet silencers, notably including those lacking REST motifs, consistently showed strong repression activities (fig. S6). These results suggest that the TREDNet framework, integrating DNase, H3K27ac, and H3K27me3 profiles, can robustly predict silencers across cell lines.

We evaluated the cross-context performance of the DLPFC TREDNet model across other brain regions—angular gyrus, anterior caudate, cingulate gyrus, hippocampus, inferior temporal lobe, and substantia nigra—and an unrelated osteoblast line. Notably, predictive accuracy (auROC and auPRC) was significantly reduced in these brain regions compared to the DLPFC (Student’s *t* test *P* < 0.0004; fig. S7), with a further decline observed in osteoblasts (*P* < 0.015 versus brain regions; fig. S7). These results indicate that the DLPFC TREDNet shows strong specificity to the DLPFC, although some DLPFC regulatory syntax is shared with other brain regions.

We next assessed the architectural sensitivity of the TREDNet framework by training three alternative phase-two models on the training data for the DLPFC TREDNet—(A) a Convolutional Neural Network (CNN)-transformer hybrid model with a comparable number of trainable parameters to the original DLPFC TREDNet, (B) a CNN model with approximately three times more parameters, and (C) a CNN model with three times fewer parameters (see Supplementary Notes). On test samples, DLPFC TREDNet predictions show strong correlations with those of models A and B (fig. S8), suggesting the resilience of the TREDNet framework to architectural variations, provided model capacity is sufficient to capture the regulatory mechanisms in the DLPFC. A clear deterioration in predictions was observed only when the number of parameters was reduced by threefold (alternative model C; fig. S8).

### AD-associated regulatory SVNs lead to substantial gene up-regulation in SL loci

**Fig. 2.**
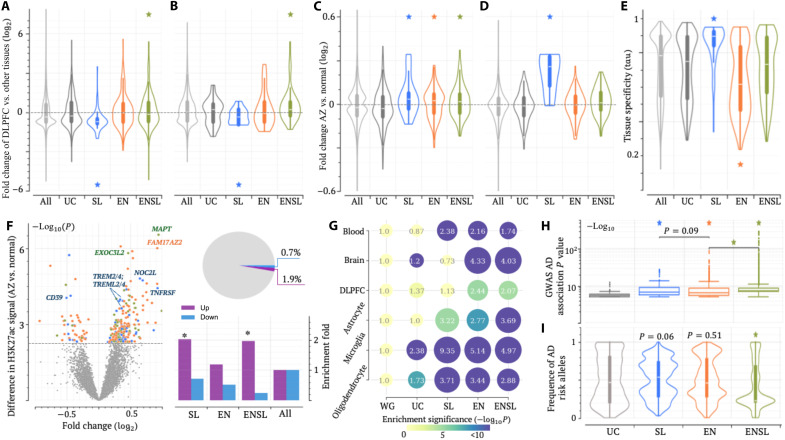
Unique transcriptomic and epigenomic patterns for each locus class. (**A** and **B**) Normalized expression levels in the healthy DLPFC for genes associated with different locus classes, linked by (A) genomic proximity and (B) brain eQTL associations. (**C** and **D**) Expression fold changes between healthy and AD DLPFCs for genes associated with different classes via (C) proximity and (D) brain eQTLs. (**E**) Tissue specificity (quantified by tau) of genes within different locus classes (see Supplementary Notes). In (A) to (E), *P* values (*P < 0.05) were determined against all examined genes using two-sided Wilcoxon rank sum tests without adjustment. (**F**) Changes in H3K27ac signals between healthy and AD DLPFCs (the volcano plot in the left) and their distribution across different locus classes (the bar plot and the pie chart in the right). In the volcano plot, each dot represents a H3K27ac peak. Gray dots indicate a peak with an insignificant change. Blue, orange, and blue dots indicate peaks with significant changes located in SL, EN, and ENSL loci, respectively. Results for H3K27me3 signals are presented in fig. S10. *P* values (*P < 10^–5^) were determined against all H3K27me3 peaks using two-sided binomial tests without adjustment. (**G**) Enrichment of chromatin contacts detected in brain and blood cells, with numbers in dots indicating enrichment levels. Dot sizes and colors denote enrichment level and significance, respectively. (**H**) Distribution of GWAS association significance (−log_10_*P*). (**I**) Frequency of AD risk alleles among radSNVs. In (H) and (I), *P* values (*P < 10^–10^) were determined against UC-locus adSNVs (*n* = 2267) using two-sided Wilcoxon rank sum tests without adjustment. In (A) to (E), (H), and (I), the center line in a box shows the median; the box bounds represent the lower and upper quartiles; the whiskers extend to the minima and maxima points up to a maximum of 1.5× the interquartile range.

To investigate the biological roles associated with AD susceptibility locus classes, we turned to the genes within these loci. Although proximal genes may not fully account for long-range regulatory targets, they are commonly used to infer the biological functions of regulatory loci (*47*). Notably, genes within SL loci exhibit lower expression in the healthy DLPFC than all assayed genes (Wilcoxon rank sum test *P* = 7 × 10^–7^) and than those in EN loci, ENSL loci, or unclassified AD susceptibility loci (represented as UC loci, *P* ≤ 4 × 10^–7^; Fig. 2A). In contrast, genes in ENSL loci are expressed at high levels (*P* = 0.04 versus all genes).

To explore regulatory effects beyond genomic proximity, we used brain cell gene-locus associations documented in two expression quantitative trait loci (eQTL) datasets: the GTEx project (*48*) and a brain single-cell eQTL database (*49*). By correlating genotypic variations with transcriptomic changes, eQTL analyses reveal regulatory interactions between genomic loci and genes. Among brain eQTL genes, those associated with SL-locus radSNVs are expressed at the lowest levels (*P* ≤ 0.03 versus other brain eQTL genes), whereas ENSL locus–associated genes exhibit the highest expression levels (*P* ≤ 0.009; Fig. 2B). These findings, along with those based on proximal genes (Fig. 2A), corroborate the silencing and activating effects of SL and ENSL locus radSNVs, respectively.

We further examined differential gene expression between healthy and AD brains (*50*). Unexpectedly, genes in either SL, EN, or ENSL loci are up-regulated more frequently in AD brains than other genes (Wilcoxon rank sum test *P* < 0.05 versus all tested or UC-locus genes; Fig. 2C). Notably, 60% of SL-locus differentially expressed genes (DEGs) are up-regulated in AD brains, surpassing the 43% of all DEGs and 53% of DEGs in all AD susceptibility loci (binomial test *P* < 10^–5^). This suggests that SL-locus genes exhibit the largest up-regulation levels, likely due to aberrant silencer activity. In further support of this hypothesis, brain eQTL genes associated with SL-locus radSNVs show the highest up-regulation in AD brains (Wilcoxon rank sum test *P* < 0.0008 versus all brain eQTL genes; Fig. 2D), with 100% of these DEGs being up-regulated (*P* < 10^–5^ versus 49 and 56% for EN-locus and ENSL-locus eQTL DEGs, respectively). Furthermore, SL-locus genes show the highest tissue specificity among all tested genes (*P* < 10^–8^), whereas ENSL-locus genes exhibit the lowest (*P* < 10^–5^; Fig. 2E). Similar patterns were also observed among target genes of SL-locus radSNVs (as defined by High-throughput Chromosome Conformation Capture (Hi-C) contacts detected in the prefrontal cortices (*17*, *51*)—these genes are lowly expressed in normal DLPFC (Wilcoxon rank sum test *P* = 0.0002 versus all examined genes) while significantly up-regulated in AD brains (*P* = 0.03^–5^; fig. S9). These findings suggest AD-protective roles to silencers in SL loci, with radSNVs deactivating silencers and leading to disease-associated overexpression of their target genes.

In AD DLPFC, gene up-regulation trends align with the alterations in histone modifications embodied by the gain of H3K27ac activity and loss of H3K27me3 activity, together symptomatic of enhancer gains and silencer losses (see Materials and Methods and table S4). For example, 3.4% of H3K27ac peaks in ENSL loci exhibit a significant intensity increase, representing a twofold enrichment compared to all H3K27ac peaks (binomial test *P* < 10^–10^). Conversely, only 0.2% of H3K27ac peaks in these regions show decreased intensities, a marked depletion compared to 1.2% of all H3K27ac peaks (*P* < 10^–10^; Fig. 2F). Similar trends are observed for SL and EN loci (*P* < 10^–5^ versus all H3K27ac). Furthermore, H3K27me3 peaks in SL and ENSL loci feature decreased intensity in AD DLPFC (*P* < 10^–5^ versus all H3K27me3 peaks; fig. S10). For example, H3K27ac signals significantly rise in AD DLPFC at the ENSL loci containing *MAPT* and *EXOC2L3* and at the SL loci hosting *TREM2* and *TREML4*. H3K27me3 signals decrease in the SL loci containing *MS4A* and *HLA-D* genes in the AD DLPFC. Combined, these results argue for combinatorial enhancer gain and silencer loss playing an additive effect in boosting AD-associated gene up-regulation.

Chromatin organization data further highlight divergent regulatory activities across locus classes. Overall, elevated levels of Hi-C contacts were observed in all three classes—SL, ENSL, and EN loci—across different brain cells, often significantly greater than the level of contacts in the genome overall (*P* < 10^–5^ versus the genome-wide average), consistent with that radSNVs of these three classes are associated with actively up- and down-regulated genes. ENSL and EN loci, enriched for enhancer radSNVs, show particularly dense chromatin contacts detected by H3K27ac HiChIP screens in brain cells (*52*) (binomial test *P* < 10^–10^ versus the entire human genome and UC loci, represented by the second row in Fig. 2G), while SL loci, primarily comprising silencer radSNVs, exhibit fewer such chromatin contacts (*P* < 10^–10^; Fig. 2G). On the other hand, this trend reverses among chromatin contacts detected in Hi-C assays not restricted to H3K27ac regions. SL loci, alongside EN and ENSL loci, are enriched with chromatin contacts detected using a Hi-C assay for prefrontal cortices (*51*). Notably, using single-cell Hi-C chromatin interactions detected for prefrontal cortices (*17*), we observed that SL loci exhibit the highest densities of these interactions in microglia and oligodendrocytes, key cell types for neuroimmune regulation (*P* < 10^–7^; Fig. 2G). This trend was also observed in blood cells (*P* < 10^–7^ SL loci versus all other loci) (*53*), key modulators of the immune system. These findings reinforce the regulatory importance of SL, ENSL, and EN locus classes for brain cells and highlight specifically elevated regulatory activity in SL loci involved in neuroimmune regulation.

radSNVs in SL, ENSL, and EN loci are located closer to their nearest transcription start sites (TSSs) than the other distal-RE (Wilcoxon rank sum test *P* < 10^–10^; fig. S11). They also exhibit stronger AD associations than those in UC loci (*P* < 10^–30^; Fig. 2H), with those in ENSL and SL loci ranking at the top. radSNVs in ENSL loci have the lowest disease allele frequencies (*P* < 10^–50^ versus other radSNVs; Fig. 2I), while those in SL loci exhibit the highest (*P* ≤ 0.06 versus other radSNVs). Collectively, while all locus classes contribute to AD pathogenesis, each class features unique transcriptomic, epigenomic, and genotypic signatures, indicating their distinct roles in this disease. Our locus annotations may offer critical insights into the molecular basis of this complex polygenic disease.

### CTCF and REL repression are among key disruptions by silencer radSNVs

To investigate regulatory circuits associated with each locus class, we analyzed the abundance of TF binding motifs mapped to radSNVs. The regulatory effect of a TF was quantified by comparing the density of its binding motifs in H3K27ac ChIP-seq peaks (indicative of regulatory activation) versus H3K27me3 ChIP-seq peaks (potentially marking repression). A binding motif enrichment in H3K27ac peaks was recorded as a positive effect score, while the enrichment in H3K27me3 peaks was recorded as a negative effect score (see Supplementary Notes). Consistent with their epigenomic features, silencer radSNVs are highly enriched for TF motifs having negative effect scores (*P* = 2 × 10^–6^ versus all TF motifs; fig. S12), while enhancer radSNVs are associated with the motifs with positive effect scores (*P* = 10^–18^; fig. S12). By contrast, adSNVs located in either H3K27ac or H3K27me3 peaks but not predicted as silencer/enhancer radSNVs show no significant bias toward either effect scores (fig. S12), further supporting the ability of the DLPFC TREDNet to identify potential silencer and enhancer variants.

Notably, TF motif enrichment profiles differ across radSNV classes (fig. S13). For example, CTCF binding motifs are enriched uniquely among silencer radSNVs in SL loci, whereas REL binding motifs are preferentially overrepresented among silencer radSNVs in ENSL loci (fig. S13). These enrichments are attenuated for AD risk alleles. For example, among silencer radSNVs, TF motifs enriched in risk alleles show elevated effect scores compared to those in protective alleles (*P* = 7 × 10^–6^; fig. S13). It suggests that substitutions at these radSNVs are associated with motif loss for repressive TFs (such as REL, TF63, SIX3, and CTCF; fig. S13) and/or the gain of activating motifs (for instance, ERG1, GLI1, and SPI1) during AD progression. This trend aligns with the pattern of gene up-regulation observed in SL loci (Fig. 2, C and D), likely due to the loss of silencing activity. Together, radSNVs across locus classes exhibit diverse sequence features, with each class recruiting a distinct set of TFs and regulatory networks. These regulatory networks are rewired during AD progression.

### SL-locus radSNVs are strongly associated with immune responses and autoimmune phenotypes

**Fig. 3.**
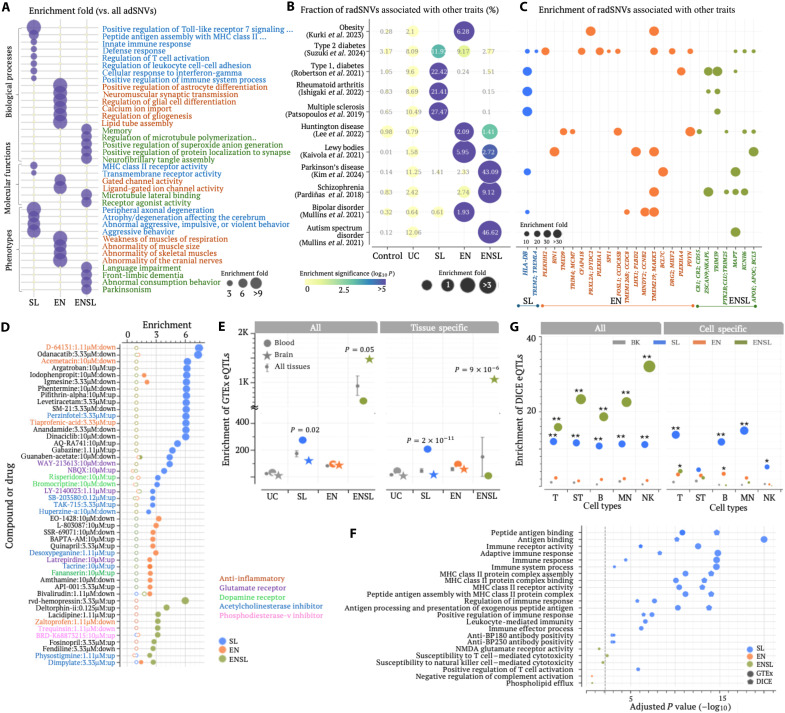
Functional distinctions across locus classes. (**A**) Functional associations (enrichment folds) of radSNVs across locus classes, based on GREAT analysis. Only significant associations are shown here. (**B**) Proportions of radSNVs associated with other diseases (%) as indicated by the numbers in dots. Dot colors indicate enrichment levels compared to all GWAS SNVs (represented as WG here). (**C**) Enrichment of radSNVs associated with other diseases across different loci compared to WG. Dot sizes indicate enrichment folds. Only significant enrichments are shown here. (**D**) Enrichment of compound-responsive genes in locus classes, with hollow dots indicating insignificant enrichments compared to genome-wide averages. (**E**) Enrichment of radSNVs across locus classes among GTEx eQTL variants in brain and blood. Silencer and enhancer radSNVs exhibit comparable enrichment levels (fig. S15). Data are presented with the median ± SEM. *P* values were determined against enrichments in all tissues (*n* = 22) using the two-sided Student’s *t* tests without adjustment. (**F**) Functional analysis of eQTL genes. (**G**) Enrichment of DICE eQTLs among radSNVs as compared to all GWAS SNVs. ST denotes simulated T cells. BK represents the adSNVs not labeled as radSNVs. *P* values (**P < 10^–5^and *P < 0.05) were determined against BK using two-sided binomial tests without adjustment. MHC, major histocompatibility complex; NMDA, *N*-methyl-d-aspartate.

To assess the biological role of radSNVs in different locus classes, we used the Genomic Regions Enrichment of Annotations Tool (GREAT) (*47*). Notably, despite slight overlap, each locus class has unique biological functions (Fig. 3A). SL loci are uniquely associated with immune-related processes, including immune defense and cellular responses to interferon-γ (IFN-γ, GREAT *P* < 10^–20^). IFN-γ, a critical proinflammatory cytokine for brain defense against latent invaders, has been implicated in microglial hyperactivation in AD brains (*54*). In contrast, EN loci are associated with lipid tube assembly, whereas ENSL loci preferentially govern memory processes and synaptic activity (Fig. 3A).

To explore the phenotypic influence of radSNVs in these locus classes, we examined their associations with 11 diseases reportedly relevant to AD. They included six neurological disorders—Parkinson’s disease (PD) (*55*), bipolar disorder (*56*), autism spectrum disorder (ASD), schizophrenia (*57*), Lewy body disease (*58*), and Huntington’s disease (*59*)—as well as three autoimmune diseases [multiple sclerosis (*60*), type 1 diabetes (*61*), and Rheumatoid arthritis (*62*)] and two metabolic conditions [type 2 diabetes (*63*) and obesity (*64*)]. Notably, more than 20% of radSNVs in SL loci are associated with all three tested autoimmune diseases, far exceeding those observed among common SNVs or other radSNVs (binomial test *P* < 10^–16^; Fig. 3B). These enrichments underscore the pivotal role of SL-locus radSNVs in immune system regulation, aligning with the results from GREAT (Fig. 3A). In contrast, ENLS-locus radSNVs are frequently associated with ASD and PD (*P* < 10^–20^ versus GWAS SNVs or other radSNVs; Fig. 3B and see Materials and Methods), two neurological disorders linked to tau pathology (*65*), which is also supported by the GREAT’s findings. Meanwhile, EN-locus radSNVs are frequently associated with Lewy body and Huntington’s diseases, with over-twofold enrichments compared to other radSNVs (*P* < 10^–5^ versus GWAS SNVs or other radSNVs; Fig. 3B). These findings demonstrate distinct phenotypic contributions of each locus class, with SL loci prominently linked to autoimmune disorders, which aligns with either altered or hyperactive immune system activity involving corresponding genes being a risk factor for AD.

To further interrogate AD genetic structures shared with other diseases, we extended this analysis to individual loci (see Materials and Methods). The SL locus encompassing *HLA-D* genes, which are essential for coordinating immune response, is highly enriched for SNVs associated with PD and tested autoimmune diseases (enrichment folds >24 versus GWAS SNVs, *P* < 10^–13^; Fig. 3C), highlighting the dysregulation of these genes as the molecular basis shared between AD and these diseases. Similarly, the ENSL locus hosting *MAPT* (encoding tau protein) is identified as a genetic link shared by AD, ASD, and PD. The ENSL *APOE* and the EN *BIN1* loci are associated with Lewy body disease, which pinpoints genetic overlaps between these dementia types (Fig. 3C).

In addition, using the brain disorder–associated genes reported by Emani *et al.* (*18*), we found that, as expected, all classes of AD susceptibility loci were enriched with AD/dementia-associated genes. Notably, SL loci were selectively enriched for AD-associated genes (*P* < 10^–10^ versus GWAS SNVs or other adSNVs; fig. S14), whereas EN and ENSL loci were more broadly linked to multiple brain disorders, a pattern consistent with our GWAS variant–based analyses (Fig. 3B). Collectively, these analyses delineate shared genetic underpinnings between AD and other diseases, offering genetic and molecular insights for further investigation.

To explore cellular responses modulated by these locus classes, we used gene expression data from the L1000 project (*66*), which catalogs genes significantly up- or down-regulated by thousands of small-molecule perturbagens in more than 200 cell types, including eight neuronal cell types. Each locus class exhibits unique perturbagen response profiles, with slight overlap among other classes (Fig. 3D). SL-locus genes are often responsive to acetylcholinesterase inhibitors (e.g., perzinfotel and huperzine-a) and glutamate receptor antagonists (such as NBQX and LY-2140023, *P* < 10^–7^ versus all tested genes), both compound classes undergoing investigation for AD treatment (*5*). EN-locus genes are enriched among those regulated by latrepirdine and tacrine, both approved for AD treatment. ENSL-locus genes are often modulated by physostigmine (another acetylcholinesterase inhibitor) and phosphodiesterase-v inhibitors (e.g., trequinsin and BRD-K68873215), a drug class with potential for AD treatment pending further validation (*67*). Furthermore, SL-locus genes are frequently down-regulated by three anti-inflammatory compounds (D-64131, acemetacin, and tiaprofenic acid, *P* < 10^–7^ versus all tested genes), with top-ranked enrichment levels (Fig. 3D), consistent with experimental anti-inflammatory strategies for mitigating AD risk (*68*).

Moreover, given the pronounced sex bias in AD risk (*69*), we examined the sex specificity of AD associations across locus classes. Both ENSL and EN loci are enriched for sex-specific AD-associated genes (*P* ≤ 0.04 versus the whole genome), consistent with enhancer-driven transcriptional dimorphism in AD (*8*). In contrast, SL loci show significant depletion of these genes (*P* = 0.01 versus the whole genome, *P* = 0.0003 versus EN or ENSL loci; fig. S14), indicating that neuroimmune pathways potentially regulated by SL loci represent a core, sex-independent mechanism underlying AD progression.

Overall, all locus classes demonstrate robust biological and pathophysiological associations with AD compared to the whole genome or UC loci, each displaying distinct functional specializations. SL loci are predominantly engaged in immune-related processes; ENSL loci are linked to tau pathology; and EN loci contribute to metabolic regulation. Our results suggest a potential for developing personalized treatment of AD based on the SL/ENSL/EN locus profiles of patients, targeting the specific pathways corresponding to a genetic passport of an individual.

### eQTLs confirm the primary roles of SL-locus radSNVs in regulating immune systems

To investigate the cellular mechanism influenced by radSNVs, we examined their colocalization with eQTLs, which capture the impact of noncoding SNVs on gene transcription in specific tissues or cell types. A significant overlap with eQTLs suggests an important regulatory role of variants under investigation. Using eQTL data from the GTEx project (*48*), which encompasses eQTLs for 24 distinct tissues (including brain and blood; see Materials and Methods), we observed a pronounced colocalization of SL-locus radSNVs with blood eQTLs (Student’s *t* test *P* = 0.02 versus all tissues). In contrast, ENSL-locus radSNVs preferentially coincide with brain eQTLs (*P* = 0.05; Fig. 3E). These enrichment trends persist when analyzing separately for silencer and enhancer radSNVs, further delineating the neurological and immune system components of AD into the identified locus classes and regulatory types (fig. S15).

To further refine our understanding of tissue-specific effects of radSNVs, we focused only on eQTLs unique to individual tissues—an analysis that separates general regulatory effects from tissue-specific regulatory interactions. This analysis reveals a further heightened enrichment of SL-locus radSNVs in blood-specific eQTLs and ENSL-locus radSNVs in brain-specific eQTLs (*P* = 2 × 10^–11^ and *P* = 9 × 10^–6^ versus all tissues for SL and ENSL loci, respectively; Fig. 3E). Although not abundant, brain eQTL genes associated with SL-locus radSNVs are primarily involved in immune-related processes, such as immune response and immune receptor activity (adjusted *P* < 10^–10^; Fig. 3F), as assessed using the g:Profiler (*70*). These genes often respond to anti-BP180 antibodies (g:Profiler adjusted *P* = 0.00004), a class of antibodies correlated with the incidence and severity of dementia (*71*). These findings, in line with analyses using the GREAT tool and GWAS associations (Fig. 3, A and B), reinforce the regulatory significance of SL-locus radSNVs in blood cells and ENSL-locus radSNVs in brain cells.

To further elucidate the regulatory impact of radSNVs on immune-associated genes and the corresponding cellular specificity, we leveraged the data from the database of immune cell (DICE) eQTLs (fig. S16) (*72*). Both ENSL- and SL-locus radSNVs show substantial enrichment for DICE eQTLs across blood cell types, including T, stimulated T, B, monocyte, and natural killer cells, with over 10-fold enrichments compared to all GWAS SNVs or other adSNVs (binomial test *P* < 2 × 10^–11^; Fig. 3G). SL-locus radSNVs (but not ENSL-locus and EN-locus radSNVs) are uniquely enriched in eQTLs specific to a single cell type, presenting a cell type–specific and centric impact of these variants. For example, these radSNVs coincide with monocyte-specific eQTLs three times more often than common SNVs or other radSNVs (*P* < 10^–17^; Fig. 3G). Moreover, DICE eQTL genes associated with SL-locus radSNVs, such as those in the *HLA-D* family, are enriched in immune regulation pathways (g:Profiler adjusted *P* < 4 × 10^–4^; Fig. 3F). These findings, mirroring those from GTEx-based and functional annotation analyses (Fig. 3A), further highlight SL-locus radSNVs as key modulators of neuroimmune systems with granular cellular specificity.

### SL-locus radSNVs are selectively linked to gene up-regulation in AD microglia

**Fig. 4.**
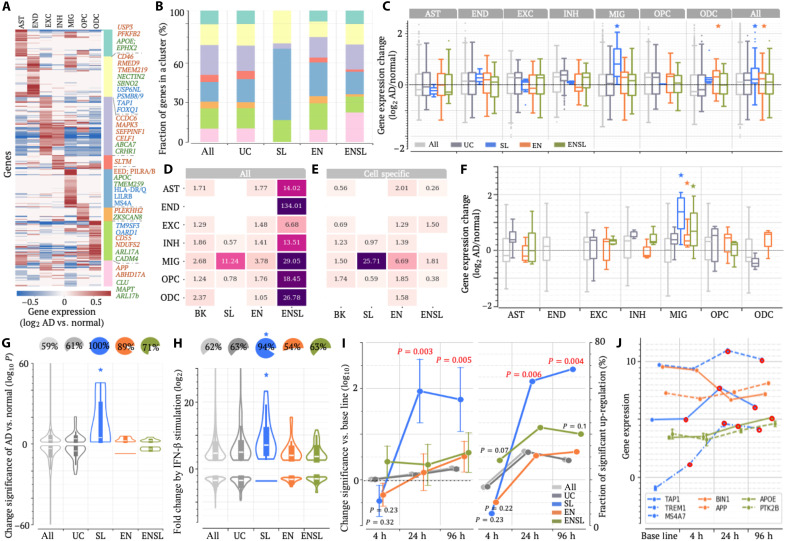
SL-locus radSNVs are associated with gene up-regulation in AD microglia. (**A**) Gene clusters based on expression changes in AD across cell types: astrocytes (AST), endothelia (END), excitatory neurons (EXC), inhibitory neurons (INH), microglia (MIC), oligodendrocyte progenitors (OPC), and oligodendrocytes (ODC). (**B**) Distribution of these clusters across locus classes. (**C**) Gene expression. *P* values (*P < 10^–5^) were determined against all examined genes (*n* = from 2884 to 4226 across individual cell types and 27601 for all cell types) by two-sided Wilcoxon rank sum tests without adjustment. (**D** and **E**) Enrichment of brain eQTLs among radSNVs for all eQTLs (D) and for cell-specific eQTLs (E). (**F**) Differential expression of microglia eQTL genes in AD microglia. *P < 10^–5^ were determined against all examined genes (*n* = from 3038 to 5540 across individual cell types) using two-sided Wilcoxon rank sum tests without adjustment. (**G**) Gene expression differences between healthy and AD microglia (quantified by differential significance −log_10_*P*, with a positive/negative value representing up/down-regulation). The pie charts display the fractions of up-regulated genes. (**H**) Gene expression after IFN-β stimulation, with the pie charts summarizing the fractions of up-regulated genes. In (G) and (H), *P < 10^–5^ were determined against all examined genes [*n* = 3629 and 951 in (G) and (H), respectively] using two-sided Wilcoxon rank sum tests without adjustment. (**I**) Differential gene expression after preformed Aβ fibril stimulations. All examined genes are the control for significance analysis. *P* values not shown are insignificant. Data are presented as median ± SEM. (**J**) Temporal gene expression profiles after the stimulation of preformed Aβ fibrils, with red-circled dots highlighting the significant up-regulations compared to untreated baseline levels. In (C), (D), (G), and (H), the center line in a box shows the median; the box bounds represent the lower and upper quartiles; the whiskers extend to the minima and maxima points up to a maximum of 1.5× the interquartile range. h, hours.

Microglia, the brain’s resident immune cells derived from monocytes, are key players in responding to harmful stimuli (*73*). The strong association of SL-locus genes with immune and hematopoietic systems, particularly monocytes, prompted an investigation into their roles in modulating microglial states and functions in healthy and AD DLPFCs. With single-cell transcriptomic data for DLPFCs from the Single-cell and Spatial RNA-Seq Database for Alzheimer’s Disease (ssREDA) data resource (*74*) and a study by Morabito *et al.* (*29*), we clustered DEGs between healthy and AD cases into eight distinct groups (Fig. 4A). Seven of these clusters exhibit cell-specific up-regulation patterns in the AD DLPFC. For example, *HLA-DRA/Q*, *EED*, *APOC*, and *TMEM529* are up-regulated exclusively in AD microglia, whereas *PTK2B* and *CRHR1* show a strong up-regulation level primarily in AD excitatory neurons. The exception cluster (the “pink” in Fig. 4A) contains the genes up-regulated across multiple cell types in the AD DLPFC, with an example of *MAPT* that is up-regulated in AD excitatory and inhibitory neurons, microglia, and oligodendrocytes.

Of SL-locus genes, 54% are up-regulated predominantly in microglia (the blue cluster in Fig. 4A), a proportion markedly surpassing the less than 25% observed for all DEGs or DEGs from other locus classes (binomial test *P* < 10^–7^; Fig. 4B). Genes in AD susceptibility loci exhibit significant up-regulation across locus classes (Fig. 4C), consistent with enhancer gains and the increase of their chromatin contacts in AD microglia (*75*). Furthermore, microglia–up-regulated DEGs in SL loci show the highest up-regulation levels (*P* = 2 × 10^–6^ versus all microglia–up-regulated DEGs; Fig. 4C). To further validate this trend, we analyzed gene expression profiles in healthy and AD microglia published by Sun *et al.* (*75*), identifying microglia DEGs. Notably, 100% of microglia DEGs in SL loci are up-regulated in AD, significantly exceeding 59% of the genome-wide average and 61 to 89% seen in other AD susceptibility loci (*P* < 10^–10^; Fig. 4D). Similarly, SL-locus up-regulated microglia DEGs exhibit the highest up-regulation levels (Student’s *t* test *P* = 6 × 10^–5^ versus other microglia–up-regulated genes; Fig. 4D).

To further evaluate the microglia specificity of SL-locus radSNVs, we used single-cell eQTLs detected across multiple brain cell types, including astrocytes, endothelial cells, excitatory and inhibitory neurons, microglia, oligodendrocytes, and oligodendrocyte progenitor cells (*49*). In contrast to EN- or ENSL-locus radSNVs, SL-locus radSNVs frequently colocalize with microglia eQTLs (binomial test *P* = 2 × 10^–15^ versus common SNVs; Fig. 4E), especially those exclusive to microglia (*P* = 10^–22^; Fig. 4F). Notably, 70% of SL-locus radSNV eQTLs are microglia specific (*P* = 0.001 versus 39% of all eQTLs; fig. S17). These findings highlight the unique microglia specificity of SL-locus radSNVs, contrasting ubiquitous roles of ENSL-locus radSNVs across brain cell types.

To assess the transcriptional impact of radSNVs in AD, we analyzed eQTL effect sizes, aligning them such that positive values denote gene up-regulation in AD brains. Notably, 90.4% of microglia eQTLs colocalizing with SL-locus radSNVs hold positive effect sizes, far exceeding 45.7% observed among microglia eQTLs (*P* < 10^–20^; fig. S18). Furthermore, microglia eQTL genes associated with SL-locus radSNVs show the highest up-regulation levels in AD microglia (Wilcoxon rank sum test *P* = 0.001 versus all microglia eQTL genes; Fig. 4G).

Similar patterns emerge when using an independent single-cell brain eQTL dataset (*18*). radSNVs co-occur with these eQTLs more frequently than the other distal-RE adSNVs (fig. S19A). SL-locus radSNVs show eQTL enrichments specific to microglia and pericytes, while EN- and ENSL-locus radSNVs are enriched with eQTLs across brain cell types (fig. S19A). Microglia-eQTL genes linked to SL-locus radSNVs are significantly up-regulated in AD microglia, mirroring the results presented above (Fig. 4, D and F).

Collectively, these findings, corroborated by both single-cell and bulk transcriptomic data (Fig. 4, B to D) and by proximal, eQTL, and Hi-C target genes, underscore the pivotal role of SL-locus genes in driving microglial dysregulation in AD and suggest that aberrant silencing of the corresponding genes in microglia is a prominent pathogenic components of AD, with microglial gene up-regulation being emblematic of AD. Of note, ENSL- and EN-locus radSNVs, both enriched for enhancer radSNVs, are associated with gene up-regulation in AD microglia (*P* < 0.001 versus all DEGs; Fig. 4, C and G, and fig. S19), albeit to a lesser extent than those in SL loci, in line with previous reports of the enrichment of AD risk variants in microglial enhancers (*76*, *77*) and increased chromatin contacts of enhancers in AD microglia (*75*).

### SL-locus genes are predominantly up-regulated during microglia inflammation

To further probe how microglia respond to inflammatory stimuli, we analyzed transcriptomic profiles in microglia-like cells generated from induced pluripotent stem cells (iMGLs), both in their basal state and following stimulation with preformed Aβ fibrils (*75*) or proinflammatory factor IFN-β (*78*). Consistent with their significant up-regulation trend in AD microglia (Fig. 4, C and F), SL-locus genes are robustly induced by IFN-β stimulation. In detail, 94% of SL-locus IFN-β DEGs are up-regulated, significantly exceeding the 64% observed in all DEGs (including those in other AD susceptibility loci, *P* < 10^–10^; Fig. 4H). Also, IFN-β–up-regulated DEGs in SL loci exhibit the highest up-regulation levels (Student’s *t* test *P* = 0.02 versus all IFN-β–up-regulated genes; Fig. 4H). Notably, 67% of SL-locus IFN-β–up-regulated DEGs are also up-regulated by preformed Aβ fibrils, representing a notable enrichment compared to 27% observed among all IFN-β–up-regulated DEGs (*P* = 0.001; fig. S20). These findings highlight the leading role of SL-locus genes in orchestrating microglial responses against diverse inflammatory stimuli.

Analyzing time-resolved transcriptomics of iMGLs exposed to preformed Aβ fibrils over the course of 4 to 96 hours (*75*), we further revealed distinct temporal patterns for different locus classes. SL-locus genes display pronounced up-regulation from 24 hours onward, contrasting with the modest early up-regulation of ENLS-locus genes at 4 hours after Aβ seeding and negligible changes of EN-locus genes throughout the course (the latter one aligns with insignificant changes between healthy and AD brains of EN-locus genes, as presented in Fig. 4C). Specifically, 28.5% of ENSL-locus DEGs are up-regulated at 4 hours after the preformed Aβ exposure (*P* = 0.07 versus 16.9% of all DEGs, *P* = 10^–5^ versus 5.3% of SL-locus genes), whereas 63.2% of SL-locus DEGs are up-regulated from 24 hours onward (*P* = 0.006 versus 32.4% of all DEGs, *P* ≤ 0.02 versus ENSL- or EN-locus genes; Fig. 4I). For example, SL-locus genes *TAP1*, *TREM1*, and *MS4A7* are up-regulated from 4 hours and beyond (Student’s *t* test, *P* < 10^–4^), whereas the EN-locus gene *BIN1* is down-regulated throughout this course (Fig. 4J). These comparisons suggest that ENSL-locus genes may respond to Aβ fibrils earlier than SL-locus genes, whereas the latter exhibit stronger up-regulation at later stages. However, experiments with finer temporal resolution will be required to confirm these dynamics. Together, different locus classes exhibit diverse cell- and time-specific response patterns during AD progression, with SL-locus genes sustaining robust up-regulation in both AD and proinflammatory-stimulated microglia.

### SL-locus genes show elevated expression levels in Aβ-predominant AD subtypes C1 and C2

The profound genetic and clinical heterogeneity of late-onset AD has driven its stratification into molecularly defined subtypes. Transcriptome analyses of hundreds of human brains have delineated five AD subtypes (A, B1, B2, C1, and C2), each exhibiting unique molecular signatures while sharing similarities in disease severity, biological sex, and the age of onset and death (*10*). Subtypes A, B1, and B2 are marked by tau protein dysregulation, with subtype A uniquely demonstrating resilience to neurofibrillary tangles. Subtypes C1 and C2 are distinguished by the overrepresentation of Aβ binding and aggregation.

**Fig. 5.**
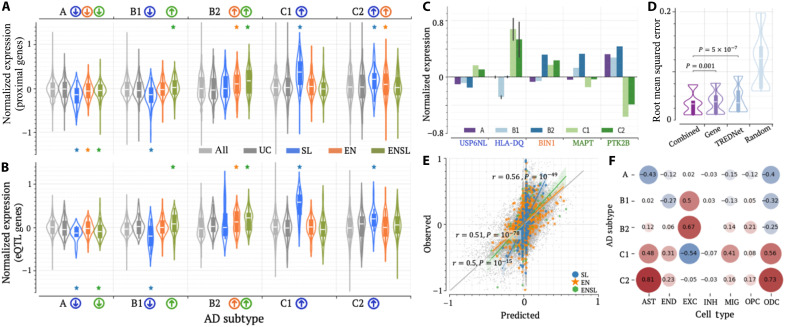
Each locus class shows a unique subtype specificity. (**A** and **B**) Subtype-specific gene expression associated with locus classes by (A) proximity and (B) brain eQTL associations. *P* values (*P < 0.03) were determined against all examined genes (*n* = 16,632) using two-sided Wilcoxon rank sum tests without adjustment. (**C**) Subtype-specific expression levels of example genes. In the case of the HLA-DQ gene family, data are presented as median ± SEM. (**D**) RMSE values calculated over test samples for linear regression models in independent 100 trials. *P* values were determined against “random” (*n* = 100) using two-sided Wilcoxon rank sum tests without adjustment. (**E**) Comparison of observed and predicted gene expression across locus classes. (**F**) Average weights in linear regression models built on cell-specific gene expression data and enhancer/silencer profiles. Cell types include astrocytes (AST), endothelial (END) cells, excitatory neurons (EXC), inhibitory neurons (INH), microglia (MIC), oligodendrocyte progenitor (OPC) cells, and oligodendrocytes (ODC). Because of the lack of gene expression in the brain immune cell type, this cell type was not included in this analysis. Weights in other models are summarized in fig. S22.

Given the functional diversity of locus classes, we examined their correspondence with AD subtypes. Genes in SL loci are overexpressed in subtype C (including C1 and C2, Wilcoxon rank sum test *P* < 0.0009 versus all genes; Fig. 5A). Subtypes B1 and B2 exhibit increased expression of genes in ENSL loci (*P* < 0.0009), whereas subtype A is characterized by low expression of genes across all locus classes (*P* < 0.03; Fig. 5A). These expression patterns were also observed among genes associated with radSNVs via brain eQTLs archived in the GTEx (*48*) or the brain cell eQTL databases (*49*). For instance, eQTL genes associated with SL-locus radSNVs are overexpressed in subtypes C1 and C2 (*P* < 0.003), whereas those with ENSL loci are up-regulated in subtypes B1 and B2 (*P* < 10^–5^). Subtype A shows down-regulation of all these genes (*P* < 0.006; Fig. 5B). For example, the genes *USP6NL* and *HLA-DQ* family, associated with SL loci by either proximity or eQTLs, exhibit elevated expression levels in subtypes C1 and C2 (Student’s *t* test *P* < 0.006 versus other subtypes). *MAPT*, an ENSL-locus gene via proximity or eQTLs, is overexpressed in subtypes B1 and B2 (*P* = 0.04; Fig. 5C).

To gain further insights into the cellular characteristics of AD subtypes, we trained Lasso regression models to predict gene expression levels for each AD subtype using cell-specific enhancer/silencer activity predictions across seven major brain cell types: astrocytes, endothelial cells, excitatory and inhibitory neurons, microglia, oligodendrocytes, and oligodendrocyte progenitor cells (see Materials and Methods). Across 100 independent trials, our models achieved an average root mean square error (RMSE) of 0.051, largely outperforming random shuffling (average RMSE = 0.12, Student’s *t* test *P* < 10^–50^; Fig. 5D) and performing comparably to models built on DLPFC cell type gene expression data (average RMSE = 0.050, *P* = 0.001). Furthermore, combining predicted enhancer/silencer profiles with cell-specific gene expression further improved regression performance (average RMSE = 0.047, *P* ≤ 0.002 versus other models). High regression accuracies were sustained across AD subtypes (fig. S21) and locus classes, with predicted expression correlating with observed expression at 0.56 for SL-locus genes (*P* = 10^–49^; Fig. 5E). Collectively, these results underscore that our enhancer/silencer profiles encompass essential regulatory components across cell types in the DLPFC, capturing the gene regulation patterns underlying different biological pathways and AD subtypes.

Furthermore, weights from the regression models were used to establish the cellular activity profiles for AD subtypes. For example, subtypes C1 and C2 correlate positively with the activity of immune-related cells, including astrocytes, microglia, oligodendrocytes, and endothelial cells, but negatively with the activity of excitatory neurons (Fig. 5F). These findings align with reports of increased microglia and astrocyte populations as well as substantial neuron loss in patients of these subtypes (*10*). They are commonly diagnosed with hyperactive astrocytosis and microgliosis in response to the excessive aggregation of Aβ. In addition, subtypes B1 and B2 show heightened excitatory neuron activity alongside moderate oligodendrocyte loss, whereas subtype A exhibits negative correlations with astrocyte and oligodendrocyte activity (Fig. 5F), supporting a distinct cellular activity profile for each AD subtype. Notably, the models built on different data types (i.e., enhancer/silencer profiles, cell-specific gene expressions, and the combination of them) show similar weights (Fig. 5F and fig. S22), validating the robustness of the TREDNet in predicting cell-specific enhancer/silencer activities, as it translates to the separation of AD subtypes.

### Deep learning identifies candidate AD causal variants by accurately quantifying their regulatory impacts

Beyond annotating the function of radSNVs, we applied the TREDNet model to assess the regulatory impact due to variants. Building on our prior study (*31*), these impacts are quantified as the difference in TREDNet-derived prediction scores between protective and risk alleles, denoted as ∆activity (see Materials and Methods). A positive ∆activity indicates that the variant increases activation or reduces repression strength, whereas a negative value reflects the opposite effect. radSNVs with significant ∆activity are considered putative causal for AD (table S3) (*31*). AD risk alleles frequently disrupt binding motifs for CTCF and generate binding motifs for the transcriptional repressor ZNF238 (*79*) at putative causal enhancer radSNVs (table S5 and Supplementary Notes), suggesting the chromatin remodeling and gain of repressor activity during AD progression, although further experimental validation will be needed to confirm these computational findings. Similarly, we observed the gain of EGR1 motifs, linked to DNA demethylation, and the loss of repressor INSM1 motifs—two TFs with key roles in brain development (*80*, *81*)—in silencer radSNVs (table S5).

**Fig. 6.**
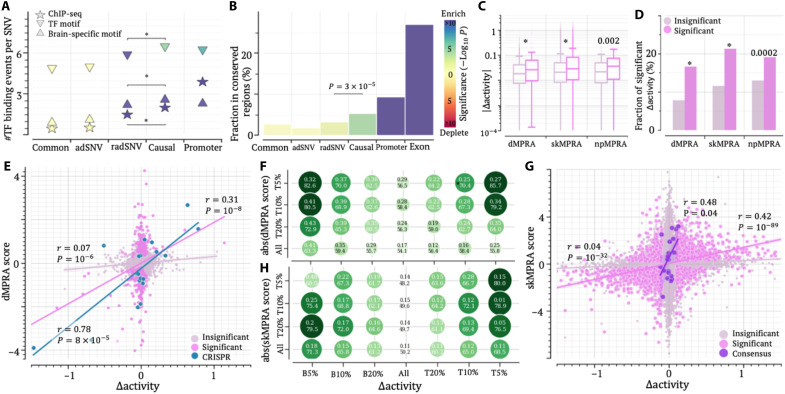
Accurate prediction of regulatory influence of SNVs. (**A**) Density of TF binding events across variant groups. Asterisks indicate significant enrichments compared to whole-genome common SNVs (WG). (**B**) Proportions of variants located in evolutionarily conserved regions, with marker and bar colors reflecting statistical significance compared to WG. In (A) and (B), *P* values (*P < 10^–5^) were determined against common SNVs using two-sided binomial tests without adjustment. Colors of markers and bars indicate the significance of P values. (**C**) Distribution of ∣∆activity∣ values and (**D**) fraction of significant ∆activity scores for the variants having significant and insignificant MPRA scores. *P* values were determined against insignificant MPRA SNVs using Wilcoxon rank sum tests without adjustment. (**E**) Correlation between ∆activity and dMPRA scores. “CRISPR” represents the variants validated in CRISPR experiments. (**F**) DCRs between dMPRA and ∆activity scores for variants, stratified by ∆activity. (**G**) Correlation between DLPFC ∆activity and skMPRA scores. “Consensus” represents the variants where skMPRA scores are directionally concordant to the MPRA measurement from other studies. (**H**) DCRs between skMPRA and ∆activity scores for variants, stratified by ∆activity. In (C) and (D), asterisks represent significant differences between significant and insignificant MPRA SNVs. In (F) and (H), the top and bottom values in a circle represent the correlation and DCRs, respectively. abs, absolute.

Identified putative causal radSNVs overlap with TF ChIP-seq peaks in the neuronal cell line SK-N-SH more frequently than common SNVs, adSNVs, or other radSNVs (binomial test *P* < 10^–10^), second only to promoter adSNVs (Fig. 6A). Similarly, these putative causal radSNVs are enriched in TF binding motifs significantly enriched in H3K27ac or H3K27me3 ChIP-seq peaks in the DLPFC (*P* < 10^–10^; Fig. 6A and see Materials and Methods). Furthermore, 5.5% of identified putative causal radSNVs reside in the regions conserved across 100 vertebrate species (*39*), significantly surpassing that for common SNVs (2.8%) or radSNVs overall (3.1%, *P* = 3 × 10^–5^; Fig. 6B). This conservation level trails only that of SNVs in exon and promoter regions, both of which are known for high evolutionary conservation due to their functional role in cellular biology.

To directly evaluate the ability of ∆activity scores to predict changes with phenotypic impact, we used MPRA results, which assess the transcriptional activity of assayed DNA sequences. Differences in MPRA outcomes between alleles measure the alteration in regulatory strength due to variants. We used MPRA data for thousands of dementia-associated SNVs (dMPRA SNVs), which correlate strongly with CRISPR-based validations in stem cell–derived brain cells despite being conducted in neuroepithelial-like human embryonic kidney 293T cells (*20*). Significant dMPRA SNVs exhibit higher absolute ∆activity (∣∆activity∣) than insignificant dMPRA SNVs (Wilcoxon rank sum *P* = 10^−5^; Fig. 6C). Of the significant dMPRA SNVs, 16.5% have significant ∆activity scores, representing a twofold enrichment compared to the 7.7% observed among insignificant dMPRA SNVs (binomial test *P* = 10^–76^; Fig. 6D). CADD scores show no significant correlation with dMPRA outputs (fig. S23), consistent with the prior finding that these scores are insignificantly correlated with experimental measurements on regulatory activities (*82*) and therefore were not examined for regulatory impact of variants below.

Critically, ∆activity scores strongly positively correlate with dMPRA outcomes. While the correlation coefficient is modest for insignificant dMPRA SNVs (*r* = 0.07, *P* = 10^–6^; Fig. 6E), it increases substantially to *r* = 0.31 (*P* = 10^–8^) for significant dMPRA SNVs and rises further to *r* = 0.78 (*P* = 8 × 10^–5^) for CRISPR-validated significant dMPRA SNVs. Overall, 63.4% of dMPRA SNVs show directional concordance between ∆activity and dMPRA scores. This directional-concordance rate (namely, DCR) escalates with the increase of ∣∆activity∣ or/and ∣dMPRA score∣, approaching ≥82.6% among variants with the top 5% of both ∣∆activity∣ and ∣dMPRA score∣ (Fig. 6F). Among 10 CRISPR-validated radSNVs, the DCR reaches 90% (table S6), consistently affirming the robustness of ∆activity scores, which thus can be used to judge the regulatory impact of sequence variants, especially at large absolute value ∆activity scores.

We further validated ∆activity using MPRA data from the neuronal cell line SK-N-SH (termed skMPRA here) published by Gosai *et al.* (*21*). Similar to dMPRA SNVs, skMPRA SNV outcomes positively correlate with ∆activity scores. Of the significant skMPRA SNVs, 21% have significant ∆activity scores, representing a twofold enrichment compared to insignificant SNVs (binomial *P* = 10^–164^; Fig. 6D). Correlation coefficients between ∆activity and skMPRA SNV scores increase from *r* = 0.04 (*P* = 10^–32^) for insignificant skMPRA SNVs to *r* = 0.42 (*P* = 10^–89^) for significant ones (Fig. 6G). The DCR with skMPRA SNV scores approaches 74.3% for significant ∆activity scores (*P* = 10^–110^ versus 52.7% of insignificant ∆activity) and further rises to 83.2% for those with ∣∆activity∣ > 0.2 (Fig. 6H). These patterns closely mirror those observed with dMPRA outcomes, reinforcing the reliability of ∆activity scores for evaluating the impact of brain variants.

Further validations using MPRA data from human neural progenitors [represented by neural progenitor MPRA (npMPRA) below] published by McAfee *et al.* (*22*) also corroborate these findings. A significant correlation of *r* = 0.54 (*P* = 0.005) and a DCR of 72.2% among significant npMPRA SNVs (fig. S24A) underscore the robustness of ∆activity scores. Consistently, ∆activity scores significantly correlated with the MPRAduo activity scores in SK-N-SH cells (*43*), with the Pearson’s *r* = 0.20 (*P* = 10^–5^) and the DCR of 55% among significant MPRAduo variants. These values further increase to *r* = 0.32 and the DCR of 64.6% among the variants having the top 5% of both ∣∆activity∣ and ∣MPRAduo score∣ (binomial test *P* = 0.0003; fig. S24B).

Furthermore, of 266 SNVs tested across multiple independent MPRA studies, 141 (53%) of SNVs have concordant direction of MPRA scores, hereafter referred to as consensus SNVs; the remaining variants were named as dissensus. Consensus SNVs show a DCR of 57.1%, which exceeds a DCR of 49.3% among dissensus SNVs (binomial test *P* = 0.006). The DCR further increases to 62.1% among the variants having significant MPRA scores (*P* = 0.04 versus dissensus; fig. S24D). Together, validations across four independent brain-related MPRA studies consistently demonstrate the reliability of ∆activity scores in capturing the regulatory effect of SNVs in brain cells, establishing ∆activity scores as a robust metric for prioritizing causal radSNVs for AD.

### Δactivity identifies rs636317, disrupting a CTCF binding event, as a putative AD causal variant in a SL locus

**Fig. 7.**
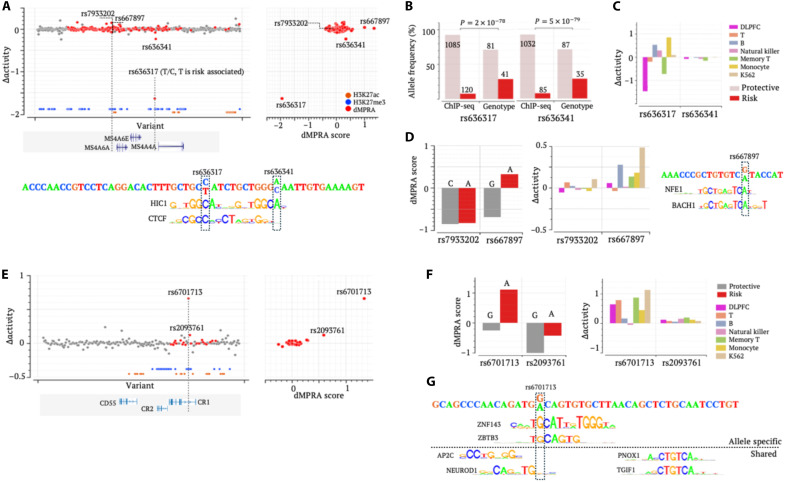
Prioritizing radSNVs in the *MS4A* and *CR1* loci. (**A**) ∆activity and dMPRA scores in the *MS4A* locus. Left: ∆activity scores; right: comparison of ∆activity (*y* axis) with dMPRA (*x* axis). (**B**) Allele frequencies for rs636317 and rs636341 in CTCF ChIP-seq reads and among samples documented in the ROSMAP project (see Supplementary Notes) (*3*). The numbers in/above bars are occurrences of alleles. *P* values were determined using two-sided binomial tests without adjustment. (**C**) ∆activity scores for rs636317 and rs636341 in the DLPFC and blood cells. (**D**) dMPRA results (left) and ∆activity scores (right) for rs7933202 and rs667897. (**E**) ∆activity and dMPRA scores in the *CR1* locus. Left: ∆activity scores; right: comparison of ∆activity (*y* axis) with dMPRA (*x* axis). (**F**) dMPRA outcomes (left) and ∆activity scores (right) for rs6701713 and rs2093761. (**G**) TF motif mapping analysis for rs6701713.

The membrane-spanning 4-domains (*MS4A*) gene cluster, for example, *MS4A6A* and *MS4A4A*, is a key modulator for immune cell activities in the brain (*83*). This genomic locus, harboring numerous adSNVs (*84*), is enriched with DLPFC silencers and is therefore categorized as an SL locus (Fig. 7A). This annotation aligns with the low expression of these genes in the healthy human brain (*85*).

Among the radSNVs in this locus, rs636317 exhibits the highest ∣∆activity∣, perfectly reflecting its leading dMPRA score among 168 dMPRA SNVs in this region (Fig. 7A). rs636317 has been marked as a likely AD causal SNV, with the allele C acting as protective and the allele T conferring AD risk. The allele C is significantly overrepresented in CTCF ChIP-seq reads (90% of reads carrying C versus its allele frequency of 67% in brain samples, *P* = 2 × 10^–78^; Fig. 7B), based on the DLPFC data documented in the ROSMAP project (see Supplementary Notes) (*3*). This result aligns with the finding that the allele T disrupts a CTCF binding site (Fig. 7A) (*30*, *84*). Despite these converging findings, the regulatory impact of the substitution at rs636317 remains unclear, as both overexpression and underexpression of *MS4A* genes have been implicated to exacerbate AD progression (*86*). Moreover, while the risk allele T is associated with reduced transcriptional activation in microglia and brain cells (as measured in dMPRA experiments), it corresponds to the increased expression of *MS4A6A* in monocytes (*84*) and the GTEx blood cells (*48*). This apparent discrepancy may reflect the pleiotropic nature of rs636317 and the context-dependent function of its host element. To probe this further, we used TREDNet models built for blood cell types (such as T and B cells, monocytes, natural killer cells, etc.) (*31*). We had previously demonstrated that ∆activity scores significantly correlate with MPRA outcomes in blood cell types, reliably prioritizing regulatory SNVs for autoimmune diseases such as type 1 diabetes (*31*). Here, rs636317 has a significantly negative ∆activity in the DLPFC but positive ∆activity scores in B cells and monocytes (Fig. 7C). These predictions are consistent with the dMPRA measurements (Fig. 7A) and eQTL associations in GTEx blood cells and macrophage (*84*), confirming the reliability of ∆activity scores, allowing us to conclude a pleiotropic influence of this variant in DLPFC and immune system cells.

rs636341, a nearby radSNV located 11 bp from rs636317, shows negligible ∆activity in both the DLPFC (consistent with its insignificant dMPRA score) and blood cells (Fig. 7C). Because of their genetic and genomic proximity, rs636317 and rs636341 show similar AD GWAS association levels and allele bias in CTCF ChIP-seq reads (Fig. 7B). This highlights the inherent difficulty in pinpointing causal SNVs amidst nearby variants and thus underscores the effectiveness of TREDNet models (broadly, deep learning models) and MPRA assays for accurately identifying causative SNVs. In addition, AD risk alleles of rs636317 and rs636341 disrupt the binding motif of HIC1 (Fig. 7A), a repressor and regulator of chromosomal stability (*87*). These disruptions potentially compromise local chromatin architecture, conferring the risk of *MS4A* dysregulation, in patients with AD. While further validation is necessary, this hypothesis offers an alternative therapeutic target for AD in the *MS4A* locus.

Last, in the *MS4A* locus, a similar trend emerges for rs667897 and rs7933202, located 53 bp apart. The risk allele at rs667897 introduces a binding site for NRE, potentially thereby augmenting *MS4A6A* expression, whereas the substitution at rs7933202 exerts an insignificant effect (*88*). Furthermore, rs667897, rather than rs7933202, has a significant dMPRA score (Fig. 7D). All these experimental assessments are successfully captured by ∆activity scores, with rs667897 holding significant ∆activity in blood cell types (such as B cell, monocytes, and K562) but the ∆activity of rs7933202 remaining insignificant across all tested blood cell types and the DLPFC (Fig. 7D).

### Candidate AD causal silencer variants in the *CR1* and *USP6NL* loci

Complement receptor 1 (*CR1* or *CD35*), a pivotal component in the innate immune system, is expressed on the surface of blood cells and facilitates the phagocytosis of immune complexes, including Aβ. The *CR1* locus, categorized as an ENSL locus, is enriched with both enhancer and silencer radSNVs. Among these variants, rs6701713 exhibits the largest ∣∆activity∣, consistent with its leading dMPRA score among 22 dMPRA variants (Fig. 7, E and F). The 60-bp-long sequence surrounding rs6701713 contains binding motifs for transcriptional repressors, including PNOX1, TGIF1, and NEUROD1 (Fig. 7G). The protective allele G at rs6701713 forms binding motifs for ZNF143, a CTCF cofactor, and ZBTB3, a chromatin looping organizer and transcription repressor (*89*). The risk allele A, disrupting these motifs, is associated with increased *CR1* expression across multiple brain regions, such as the frontal cortex and hippocampus (fig. S25). These findings support the rs6701713-hosting element as a silencer, maintaining low expression of *CR1* in the healthy brain. Disruption of this silencer by the G-to-A substitution at rs6701713 is associated with *CR1* up-regulation in AD (*90*). All reports support a significant positive ∆activity at rs6701713. Our motif analysis further suggests that this regulatory alteration is probably due to the loss of ZNF143 binding.

In the *USP6NL* locus, another SL locus, seven SNVs were probed in dMPRA experiments. Of them, rs7920721 corresponds to the most significant dMPRA score and ∆activity (fig. S26). The risk allele G at rs7920721 weakens the binding motif for NEUROG1, a TF essential for brain development. In addition, rs12359970, a variant not examined in the dMPRA, exhibits the highest ∆activity in this locus. The AD risk allele G at this variant disrupts a binding motif of HMGA2—a TF involved in neuron development and AD pathogenesis (*91*), suggesting the potential contribution of this variant to the dysregulation of *USP6NL* in AD.

### Complexity of AD is reflected in combined impact of silencer and enhancer putative causal variants

*PTK2B*, a nonreceptor tyrosine kinase, is one of the few validated genes for late-onset AD, with diverse roles in neuroinflammation, neuronal development, and synaptic plasticity (*92*). *PTK2B* down-regulation is linked with tau hyperphosphorylation, whereas its overexpression is associated with Aβ-induced phenotypes such as memory impairment and synapse loss (*92*, *93*). The *PTK2B-CLU* locus, enriched with enhancer and silencer radSNVs, is categorized as an ENSL locus.

**Fig. 8.**
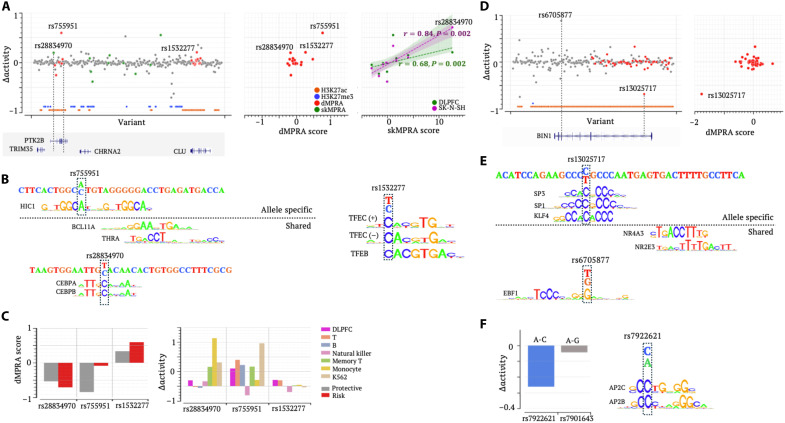
Prioritizing radSNVs in additional AD susceptibility loci. (**A**) ∆activity and dMPRA scores in the *PTK2B-CLU* locus. Left: ∆activity scores; middle and right: comparisons of ∆activity scores with dMPRA and skMPRA results, respectively. (**B**) TF motif mapping analysis for rs755951, rs28834970, and rs1532277. (**C**) dMPRA results (left) and ∆activity scores (right) for rs755951, rs28834970, and rs1532277. (**D**) ∆activity and dMPRA scores in the *BIN1* locus. (**E**) TF motif mapping analysis for rs13025717 and rs6705877. (**F**) ∆activity scores for rs7922621 and rs7901634 in the DLPFC.

Of 18 probed radSNVs, dMPRA experiments identified three significant variants. Two of them—rs755951 and rs1532277—reach significant ∆activity scores. rs755951 displays the highest ∆activity and dMPRA scores (Fig. 8A). Its risk allele C, corresponding to increased *PTK2B* expression in blood cells (fig. S25), disrupts, at least attenuates, the binding motif of HIC1, a transcriptional repressor and chromatin organizer (Fig. 8B). Another significant dMPRA SNV is rs1532277, where the C-to-T substitution introduces binding motifs for TFEC and TFEB (Fig. 8B), transcriptional activators associated with neurodegenerative disorders, including AD (*94*).

Another notable radSNV, rs28834970, resides in an intronic enhancer of *PTK2B* in neuronal cells. This variant is associated with increased *PTK2B* expression in blood cells and an elevated abundance of phosphorylated tau in cerebrospinal fluid (*93*). Although insignificant in dMPRA, this variant shows significant positive ∆activity scores in the DLPFC and SK-N-SH cells, supported by a significant positive skMPRA score (Fig. 8A). In blood cells (such as monocytes), the T-to-C substitution at this variant has significantly positive ∆activity scores (Fig. 8C), consistent with increased *PTK2B* expression in blood cells (fig. S25). These positive ∆activity scores are further supported by the finding that the risk allele T introduces binding motifs of the activator CEBP family (Fig. 8B). All these validate ∆activity predictions at this variant in the DLPFC and blood cells. In this locus, SK-N-SH ∆activity scores show a stronger correlation with skMPRA (*r* = 0.84, *P* = 0.0002) than the DLPFC ∆activity scores (*r* = 0.68, *P* = 0.0002; the third panel in Fig. 8A), showcasing the ability of TREDNet models to capture cell-specific regulatory signatures.

Additional examples of identified putative causal radSNVs are in the *BIN1* locus, an EN locus. Among 51 dMPRA-tested radSNVs, rs13025717 shows the lowest ∆activity, coinciding with its lowest dMPRA score (Fig. 8E). The C-to-T substitution at this SNV reduces the ATAC-seq signal and disrupts the binding motifs for SP1 and KLF4, a transcriptional activator and partner of PU.1 (Fig. 8F) (*84*). These together validate the significant negative ∆activity score. Another identified putative AD causal variant in this locus is rs6705877, an intronic SNV not tested in MPRA experiments. The risk allele T at this variant generates a binding motif for EBF1 (Fig. 8E) and is associated with increased *BIN1* expression, as reported in the GTEx data (fig. S25). With these findings, rs6705877, carrying the most significant ∆activity in the *BIN1* locus, is a strong candidate for further investigation.

In the *TSPAN14-MAT1A* locus, two proximal SNVs—rs7922621 and rs7910643, located 225 bp apart—reside within the same intronic enhancer (*78*). Genome editing experiments confirmed rs7922621, rather than rs7910643, as a putative causal variant for the down-regulation of *TSPAN14* (*78*). These results are accurately reflected by ∆activity = −0.262 and −0.042 for rs7922621 and rs7910643, respectively. The C-to-A substitution at rs7922621 disrupts binding motifs for AP2C and AP2B (Fig. 8F), providing mechanistic insights into its regulatory effect.

## DISCUSSION

The escalating global prevalence of AD, coupled with its pronounced heritability, has spurred decades of research into the genetic architecture of this disease. Despite significant advancements, inadequate annotation of regulatory SNVs associated with AD (adSNVs) remains a major obstacle to progress. To address this gap, we developed a deep learning framework, TREDNet, to predict the regulatory impact of these variants. Using this approach, we detected 1457 silencer and 3084 enhancer AD variants (collectively termed radSNVs) in DLPFC. radSNVs show stronger AD associations reported in GWASs when compared to the general pool of adSNVs. Silencer radSNVs are predominantly associated, via proximity or eQTLs, with genes lowly expressed in the healthy DLPFC, whereas enhancer radSNVs are associated with highly expressed genes.

Assessing the regulatory influence of radSNVs is essential for elucidating the genetic underpinnings of AD. Nucleotide-resolution tools such as MPRA assays and machine learning models are instrumental in this pursuit, offering nucleotide-level insights into regulatory mechanisms (*20*–*22*, *95*, *96*). However, as prior studies have noted, MPRA assays often exhibit biases. Their low sensitivity of experimental constructs and signal-calling strategies to silencing sequences results in an overrepresentation of SNVs with at least one activating allele (*31*, *97*). For example, in skMPRA, 13,566 variants carry H3K27ac signals in the DLPFC, while only 3731 variants show H3K27me3 modification. Similarly, in dMPRA, 1612 and 207 variants overlap with H3K27ac and H3K27me ChIP-seq peaks in the DLPFC, respectively. In this context, machine learning models present a promising alternative. Our TREDNet model, which explicitly quantifies silencer and enhancer activity by analyzing DNA sequence composition, demonstrates robust predictive power of silencers and enhancers, as well as their disruptive variants, as evidenced by significant correlations with experimental results from MPRAs and beyond. Applying this framework, we identified putative causal radSNVs for AD. These variants are characterized by notable enrichment in neuronal cell TF ChIP-seq peaks, brain-specific TF binding motifs, and evolutionarily conserved regions, confirming their pivotal regulatory roles.

The distribution of radSNVs categorizes AD susceptibility loci into three classes—12 SL loci, 41 EN loci, and 12 ENSL loci. Each class, exhibiting unique transcriptomic and epigenomic patterns during AD progression, is associated with specific biological functions, molecular activities, and perturbation responses. For example, genes associated with SL loci are suppressed in the healthy DLPFC but up-regulated in the AD DLPFC, accompanied by reduced H3K27me3 and increased H3K27ac levels in patients with AD. EN-locus genes often govern metabolic processes, such as gated channel activities and lipid tube assembly, and are modulated by channel blockers such as BAPTA-AM and quinidine. ENSL loci are chiefly associated with tau-related pathways and synapse degeneration. In contrast, SL loci are pivotal for neuroimmune response and microglia regulation. Moreover, each locus class has unique phenotypic associations. SL radSNVs are genetic components frequently shared by AD and autoimmune disorders; ENSL radSNVs are common genetic factors for both AD and tau-associated neurological diseases, such as PD; EN loci are exclusively associated with Lewy bodies. Collectively, by categorizing AD susceptibility loci, we delineate the complex regulatory landscape of AD into distinct biological pathways, providing regulatory insights for its pathogenesis.

Our silencer and enhancer profiles were also applied to illuminate molecular and cellular features of five main AD subtypes. Using them, we reported that Aβ overaccumulation subtypes are characterized by SL-locus gene up-regulation, overreactive microglia and astrocytes, and excitatory neuron loss. Tau pathology–dominant subtypes are marked by up-regulated ENSL-locus genes and hyperactive excitatory neurons. These findings align with experimental observations, validating the contribution of our framework and providing previously unknown avenues for the mechanistic interpretation of AD phenotypes across different patient cohorts.

We demonstrated here the increased H3K27ac intensity in AD DLPFCs, the significant up-regulation of ENSL-locus genes in Aβ-exposed iMGLs (*75*), and the elevated expression of enhancer radSNV–associated eQTL genes in AD microglia (*49*). In line with prior reports of AD risk variant enrichment in microglial enhancers (*76*, *77*) and widespread enhancer contact gain in microglia (*75*), our findings reinforce the critical role of microglial enhancers in AD pathogenesis. Our analyses extended beyond enhancer-associated effects, revealing that SL loci—enriched with silencer AD variants—exert distinct and significant contributions to transcriptional dysregulation in AD microglia. More than half (54.2%) of SL-locus genes are up-regulated in AD microglia, representing a fourfold enrichment compared to the genome-wide average. Moreover, 70% of SL-locus radSNV eQTLs are microglia specific, far exceeding the 39% observed for all brain eQTLs. Notably, 90% of these eQTLs have AD risk alleles associated with gene up-regulation, representing a significant enrichment compared to all microglia eQTLs. SL-locus genes are often induced by both IFN-β and preformed Aβ fibrils in iMGL cells. These findings underscore SL-locus radSNVs as a core modulator for aberrant microglial response, a defining feature of AD pathology (*98*). We further pinpoint the potential causal radSNVs for this abnormality. For example, the silencer SNVs rs667897 and rs636317, which disrupt the binding motifs of CTCF and NFE, are likely drivers for the dysregulation of *MS4A* genes in the AD DLPFC.

The DLPFC TREDNet model relies primarily on H3K27me3 ChIP-seq peaks to predict silencers. While this histone mark is a widely accepted proxy of regulatory repression (*44*, *99*, *100*) and MPRAduo data support the accuracy of silencer predictions, our silencer profile may remain incomplete due to the existence of non-H3K27me3 silencers (*41*, *45*, *101*). Alternative silencer-associated histone markers, such as H3K79me2 and H4K20me1 (*41*, *45*), represent an additional path to explore for comprehensive mapping of disease-associated silencer variants. Nevertheless, our analyses pinpoint the distinct and critical role of silencers in modulating biological pathways underlying AD susceptibility. Looking ahead, our deep learning model can be expanded to incorporate additional functional AD data. For example, by adding spatial single-cell ATAC-seq data, our model can mark silencers and enhancers across different DLPFC and other brain regions, facilitating the investigations into brain spatial-specific attributes for AD subtypes.

In addition, although many radSNVs highlighted here were supported by previous experimental studies, underscoring the robustness of TREDNet predictions, functional validation of additional predictions remains an important next step. Systematic experimental testing of uncharacterized radSNVs reported in this study, in physiologically relevant models, will be crucial to reveal regulatory mechanisms of AD risk.

In summary, by mapping enhancers and silencers in the DLPFC, this work advances the understanding of the molecular basis of AD. Despite being only half as abundant as enhancer radSNVs, silencer radSNVs exert substantial and unique influences on AD pathogenesis, particularly through their unique source of variance and distinguishing phenotypic impact on SL loci. As silencer variants have also been implicated in different diseases, such as breast cancer (*102*), schizophrenia (*103*), and facial paresis (*104*), yet remain largely underexplored, our work presents a framework of silencer variant analysis, which can be readily extended to other diseases. Systematic investigation of silencer variants is essential for a comprehensive understanding of the genetic architecture underlying complex diseases like AD.

## MATERIALS AND METHODS

### The DLPFC TREDNet model training

We modified the TREDNet model, a two-phase deep learning model (*28*), to predict enhancers and silencers in the DLPFC and eight major cell types. We downloaded DNase-seq peaks, H3K27ac, and H3K27me3 ChIP-seq peaks (“narrow peak”) in the DLPFCs of 20 undemented postmortem brains cataloged in the Rush Alzheimer’s Disease Study (https://encodeproject.org/brain-matrix/?type=Experiment&status=released&internal_tags=RushAD). ENCODE ChIP-seq datasets used here pass ENCODE’s stringent quality control metrics, with high signal-to-noise ratios [Relative Strand Cross-correlation (RSC) > 0.8], strong peak reproducibility across replicates [Irreproducible Discovery Rate (IDR) < 0.05], and sufficient sequencing depth (>20 million uniquely mapped reads for TFs; >45 million for histone marks) (*105*). For each sample, candidate enhancers were defined as the DNase-seq peaks overlapping H3K27ac ChIP-seq peaks but lacking H3K27me3 signal within the central 400 bp. Candidate silencers were the DNase-seq peaks carrying H3K27me3 signal but lacking H3K27ac in the central 400 bp, together with H3K27me3 peaks overlapping neither DNase-seq nor H3K27ac peaks. Each of these elements was then extended to a 1-kbp sequence centered at its midpoint. To ensure the specificity for silencers and enhancers, the elements overlapping with promoters and exons of protein-coding genes annotated in the GENCODE project (*27*) were excluded. After merging data from different samples, redundancy across elements was addressed by randomly retaining an element among those overlapping with each other by more than 600 bp. This strategy aligns the midpoints of input sequences with those of original ChIP-seq peaks, avoiding potential artificial peak shifts that could occur with deterministic merging strategies (e.g., sliding windows or recentering) and thereby preserving intrinsic regulatory signals potentially captured in the central regions of ChIP-seq peaks.

To annotate the cell-specific functions of candidate elements, we integrated single-cell ATAC-seq data from three studies (*18*, *29*, *30*), encompassing chromatin accessibility profiles for eight brain cell types, in our deep learning model. These single-cell ATAC-seq peaks were detected in >70,000 cells from at least 10 individuals, with high reproducibility (*18*, *29*, *30*).

To this end, our model profiles a set of predicted enhancers and silencers for nine cellular contexts: the DLPFC and eight distinct brain cell types. Control samples were generated by randomly sampling DNase-seq peaks documented for nonbrain biosamples in the ENCODE project (*33*), excluding those overlapping with candidate silencers and enhancers, as well as epigenomic peaks used to define these elements.

To predict enhancers and silencers for nine cellular contexts, the TREDNet model consists of 19 output nodes with the activation function of “SoftMax,” corresponding to silencers and enhancers for nine cellular contexts, as well as controls. For each cellular context, the cost function was the “categorical cross entropy” of three classes—enhancer, silencer, and control. The overall cost function for training this 19-output TREDNet model was the sum of these context-specific categorical cross entropy functions. For consistency throughout the training process, sequences in chromosomes 7 and 8 were held out from all training stages (i.e., the training of both the phase-one and phase-two models) and were reserved exclusively for testing. The details of this model are as follows:

1) One-dimensional (1D) convolutional layer with 64 kernels, each having a window size of four and a step size of one.

2) Maxpooling layer with a window size of three and a step size of two.

3) Dropout layer with a dropout proportion of 0.2.

4) 1D convolutional layer 128 kernels, each having a window size of three and a step size of one.

5) Dropout layer with a dropout proportion of 0.2.

6) Fully connected layer of 100 neurons with the sigmoid activation function.

7) Fully connected layer of 50 neurons with the sigmoid activation function.

8) For each cellular context, a fully connected output layer of 3 neurons with the SoftMax activation function. Nine cellular contexts include DLPFC, astrocyte, endothelia, inhibitory neuron, excitatory neuron, immune cell, microglia, oligodendrocyte, and oligodendrocyte progenitor.

This model was trained on four NVIDIA A100 Graphics Processing Units(GPUs) [80 GB Video Random Access Memory (VRAM)]. Further technical specifics about training and other reference models are detailed in Supplementary Notes.

### Predicting silencer and enhancer variants in the DLPFC TREDNet

We collected AD-associated variants from three studies (*15*, *16*, *26*). The AD association of a SNV is the minimum of AD association *P* values across these studies. SNVs are considered as AD-associated if their AD associations are less than 10^−5^, dubbed as adSNVs. To evaluate the regulatory activities of a noncoding no-promoter adSNV, we used the 1-kbp sequence centered at this variant as the input to the TREDNet model. The outputs of the TREDNet model are our prediction scores of regulatory activities of input variants. Variants overlapping H3K27me3 peak detected in the DLPFC (as documented in the ENCODE RUSH project) were predicted as functional silencer variants if their silencer prediction score exceeded the threshold *c*_*s*,*i*_ in at least one cellular context (where *i* represents a cell context). In a cellular context *i*, *c*_*s*,*i*_ was the silencer prediction score corresponding to a false-positive rate (FPR) of 0.05 among test samples wherein 90% were controls. Conversely, variants overlapping DLPFC H3K27ac peaks were predicted as enhancer variants if their enhancer prediction score was greater than the cutoff *c*_*e*,*i*_ in at least one cellular context. In a cellular context *i*, *c*_*e*,*i*_ was the prediction score corresponding to the FPR of 0.05 among a test sample set in which 90% were controls. Furthermore, to identify regulatory adSNVs outside promoters and H3K27ac/H3K27me3 peaks, we examined adSNVs within DNase-seq peaks but carrying neither H3K27me3 nor H3K27ac signals. Of them, variants were predicted as silencer variants if their silencer scores exceeded *c*_*s*,*i*_ in at least one context and their enhancer scores were below *c*_*e*,*i*_ in all contexts; conversely, variants were classified as enhancer variants when their enhancer scores exceeded *c*_*e*,*i*_ in at least one context and their silencer scores were below *c*_*s*,*i*_ in all contexts.

### Δactivity of regulatory variants

As detailed in our prior study (*31*), the regulatory difference between two alleles for a given variant is measured as

$$∆\text{activity}=\left({\text{log}}_{10}{\text{FPR}}_{e,\text{mu}}-{\text{log}}_{10}{\text{FPR}}_{e,\text{wt}}\right)-\left({\text{log}}_{10}{\text{FPR}}_{s,\text{wt}}-{\text{log}}_{10}{\text{FPR}}_{s,\text{mu}}\right)$$

where wt and mu represent the wild-type and mutant alleles, respectively. FPR_e_ is the FPR for enhancer prediction scores among a sample set comprising 90% controls. Similarly, FPR_s_ is the FPR for silencer prediction scores. A positive ∆activity indicates a mutation-driven increase in activation impact potential. A ∆activity level was considered as significant if it falls within the top 5% (for a positive ∆activity) or bottom 5% (for a negative ∆activity) of scores among all GWAS SNVs.

### Categorization of AD susceptibility loci

We integrated AD-associated variants from three recent GWASs (*15*, *16*, *26*), resulting in a set of 18,826 adSNVs distributed across 1475 gene loci. A gene locus encompasses the gene body and flanking upstream and downstream intergenic regions. After merging adjacent gene loci hosting adSNVs, we retrieved 124 genomic regions enriched with adSNVs compared to the genome-wide average (*P* < 0.003; table S1). These regions are referred to as AD susceptibility loci. Hence, each locus probably spans multiple gene loci. We used gene annotations cataloged in the GENCODE project (*27*) to categorize AD-associated variants into two broad classes: promoter/exon and distal-RE variants. The enrichment of promoter/exon variants within an AD susceptibility locus (say *j*) was evaluated as

$$P_{j,\text{ep}}=\sum_{m=n_{\text{ep},j}}^{N}\left(\begin{array}{c} N\\ m \end{array}\right){\gamma_{0}}^{m}{\left(1-\gamma_{0}\right)}^{N-m}$$

where $\gamma_{0}$ is the ratio of the AD variants located within promoter or exon regions in the human genome, and $n_{\text{ep},j}$ is the number of promoter/exon variants in the locus *j*. Using a loose criterion *P*_*j,ep*_ < 0.05, we identified 24 AD susceptibility loci as promoter/exon variant enriched.

To evaluate the enrichment of silencer radSNVs within an AD susceptibility locus (say *j*), we compared their density to that across the entire genome

$$P_{j,S}=\sum_{m=n}^{N}\left(\begin{array}{c} N\\ m \end{array}\right){\pi_{0}}^{m}{\left(1-\pi_{0}\right)}^{N-m}$$

where $\pi_{0}$ is the ratio of the locus length to the human genome size. *N* is the total number of silencer radSNVs, and *n* is the number of silencer radSNVs in the locus *j*. The locus *j* was defined as enriched for silencer radSNVs when $P_{j,S}<0.005$. Similarly, the locus *j* is enriched for enhancer radSNVs when $P_{j,E}<0.005$. Loci enriched exclusively for silencer radSNVs were classified as silencer (SL) loci, those enriched exclusively for enhancer radSNVs as enhancer (EN) loci, and those enriched for both as enhancer-silencer (ENSL) loci.

### Enrichment of GWAS SNVs among radSNVs in individual loci

To investigate the overlap of radSNVs with GWAS SNVs associated with a disease (*d*) in a given locus (*j*), we used the genome-wide expectation as the control. That is

$$P_{j}^{d}=\sum_{k=q}^{Q}\left(\begin{array}{c} Q\\ q \end{array}\right){\omega_{d}}^{q}{\left(1-w_{d}\right)}^{Q-q}$$

where *Q* represents the number of radSNVs in the locus *j*, and *q* is the number of these radSNVs associated with the disease *d*. Here, $w_{d}$ denotes the fraction of the common variants associated with the disease *d*. Enrichment was considered significant if $P_{j}^{d}<0.005$.

### Differential signal analysis for H3K27ac and H3K27me3 modification between healthy and AD DLPFCs

H3K27ac and H3K27me3 ChIP-seq data (narrow peak and bam files) were downloaded from the RUSH project (https://encodeproject.org/brain-matrix/?type=Experiment&status=released&internal_tags=RushAD). We merged narrow ChIP-seq peaks as histone modification regions and then used DESeq2 (*106*) to evaluate epigenetic signal difference between healthy and AD cases in these merged peaks. Using *P* < 0.005, 4499 H3K27ac peaks and 2799 H3K27me3 peaks were identified to exhibit significant intensity difference (table S4). To address long-distance chromatin interactions, a histone modification region was associated with an AD susceptibility locus within 1 Mpb to its center.

### Predicting gene expression in AD subtypes using linear regression

To investigate the associations between AD subtype and brain cell types, we used a linear regression model to predict AD subtype gene expression using enhancer/silencer and gene profiles of seven brain cell types. Differential gene expression profiles between healthy and AD brains were sourced from publicly available datasets, i.e., the ssREDA resource (*74*) and a dataset from the study of Morabito *et al.* (*29*). Enhancer/silencer profiles were established by applying the TREDNet model to single-cell ATAC-seq peaks from three recent studies (*18*, *29*, *30*). ATAC-seq peaks were extended to 1 kb at their midpoints and used as the TREDNet input. The regulatory influence of an ATAC-seq peak (say *i*) in a cell type *a* (${\text{RI}}_{s}^{c}$) is measured as

$${\text{RI}}_{i}^{c}=I\left(E_{i}\right){\text{log}}_{10}{\text{FPR}}_{i,E}^{c}-I\left(S_{i}\right){\text{log}}_{10}{\text{FPR}}_{i,s}^{c}$$

where ${\text{FPR}}_{i,s}^{c}$ is the FPR for its silencer prediction score and ${\text{FPR}}_{i,E}^{c}$ is the FPR for its enhancer prediction score. $I\left(E_{i}\right)=1$ if the ATAC-seq peak *i* overlaps with a H3K27ac peak in the DLPFC and $I\left(E_{i}\right)=0$ otherwise. $I\left(S_{i}\right)=1$ if the ATAC-seq peak *i* overlaps with a H3K27me3 peak in the DLPFC and $I\left(S_{i}\right)=0$ otherwise.

Regulatory domains for a gene include the genomic regions surrounding its TSS with 200 kbp and its whole intronic region. In the cell type *c*, the regulatory impact on this gene was quantified as the sum of ${\text{RI}}_{i}^{c}$ among ATAC-seq peaks located within its regulatory domain. Furthermore, to normalize contribution across cell types and data types, gene expression and regulation impact values were scaled to a maximum of 1 within each cell type and data type.

We applied the Lasso linear model (from the Python scikit-learn library, with the setting of alpha = 0.0001, precompute = True, max_iter = 1000 and selection = “random”) to predict AD subtype gene expression (*10*) using three input types: (i) cell type differential gene expression profiles between healthy and AZ DLPFCs from the ssREAD resource (*74*), (ii) cell type regulatory impact profiles in brain as we predicted here, and (iii) a combination of both. Model performance was evaluated under a 10-fold validation, averaging RMSEs across 100 trials for each input type (Fig. 5D). Model weights, which can be interpreted as the correlations between cell types and AD subtypes, were derived as the average weights across all built models. For the models trained on the combination of gene expression and enhancer/silencer profiles, the weights are the average of the weights from different data types.

### Publicly available data and tools used in this study

The Genotype-Tissue Expression Project used here is the GTEx database version 8 (*48*), documenting eQTLs for 13 brain regions and the whole blood. All brain eQTLs were merged into an eQTL set for brain tissue. An eQTL (i.e., a variant and its associated gene) was considered tissue specific if it was detected only in a single tissue. Single-cell eQTLs for brain cell types were downloaded from two independent studies (*18*, *49*). Similarly, a brain eQTL was considered cell specific if it was only detected in a single brain cell. Blood DICE eQTLs (*72*) were sourced at https://dice-database.org/downloads. To ensure high cell specificity of DICE eQTLs, we clustered DICE blood cell types based on the similarities of their eQTL profiles, resulting in five groups of DICE cell types (fig. S16). A DICE eQTL was considered cell specific if it was only detected in a single cell type group.

We collected (i) GWAS SNVs curated in the National Human Genome Research Institute (NHGRI) catalog and in the UK Biobank release 2 cohort and (ii) the SNVs in tight linkage disequilibrium (LD *r*^2^ > 0.8) to these SNVs based on the EUR population in the 1000 Genomes Project (*31*) as the control for analyzing the association of radSNVs with other disorders and the enrichment of radSNVs among eQTL SNVs (Fig. 3, C, E, and G). Here, using GWAS SNVs, rather than all common SNVs, is to address the potential distribution bias of SNVs examined in GWAS and eQTL studies.

TF ChIP-seq data for SK-N-SH cells were downloaded from the ENCODE project. TF binding motifs were retrieved from the MEMEMultiple Expectation maximizations for Motif Elicitation (MEME) Suite (https://meme-suite.org/meme/db/motifs). The Find Individual Motif Occurrence, with the default setting, was used to map motifs in genomic sequences with the default setting.

We downloaded the 100-way evolutionarily conserved elements from the UCSC genome data resource at https://hgdownload.soe.ucsc.edu/goldenPath/hg38/database/. Cell-specific compound-responsive gene lists were from the L1000 project at https://maayanlab.cloud/sigcomlincs/#/Download. H3K27ac HiChIP chromatin contact data were retrieved from the HiChIPdb (*52*) at https://health.tsinghua.edu.cn/hichipdb/about.php. DESEQ2 was used to detect the H3K27ac and H3K27me3 ChIP-seq peaks having significantly different signals between healthy and AZ DLPFCs.

## Supplementary Materials

### The PDF file includes:

Supplementary Notes Figs. S1 to S26 Legends for tables S1 to S6
