# Supplementary material for: Silencer variants are key drivers of gene up-regulation in Alzheimer’s disease
Source: Sci Adv. 2026 Feb 11;12(7):eadz3323. doi: 10.1126/sciadv.adz3323 (PMC12893287; doi:10.1126/sciadv.adz3323)
Supplement: Supplementary file 1 — Supplementary Notes Figs. S1 to S26 Legends for tables S1 to S6 [file sciadv.adz3323_sm.pdf]

Science Advances
AAAS

Supplementary Materials for

Silencer variants are key drivers of gene up-regulation in Alzheimer's disease

Di Huang and Ivan Ovcharenko

Corresponding author: Ivan Ovcharenko, ovcharen@nih.gov

Sci. Adv. **12**, eadz3323 (2026)
DOI: 10.1126/sciadv.adz3323

The PDF file includes:

Supplementary Notes
Figs. S1 to S26
Legends for tables S1 to S6

Other Supplementary Material for this manuscript includes the following:

Tables S1 to S6

Supplementary Notes

Architectures of deep learning models

The phase-two model in the DLPFC TREDNet consists of two convolutional layers and three fully-connected layers arranged sequentially. The details of this model are:

1. 1-dimensional (1D) convolutional layer with 64 kernels, each having a window size of four and a step size of one.
2. Maxpooling layer with a window size of three and a step size of two.
3. Dropout layer with a dropout proportion of 0.2.
4. 1D convolutional layer 128 kernels, each having a window size of three and a step size of one.
5. Dropout layer with a dropout proportion of 0.2.
6. Fully connected layer of 100 neurons with the sigmoid activation function.
7. Fully connected layer of 50 neurons with the sigmoid activation function.
8. For each cellular context, a fully connected output layer of 3 neurons with the SoftMax activation function. Nine cellular contexts include DLPFC, astrocyte, endothelia, inhibitory neuron, excitatory neuron, immune cell, microglia, oligodendrocyte, and oligodendrocyte progenitor.

We compared this model with three alternatives – (A) a CNN-transformer hybrid model with a comparable number of trainable parameters to the original DLPFC TREDNet, (B) a CNN model with approximately three times more parameters, and (C) a CNN model with three times fewer parameters.

The details of the model (A) are:

1. 1-dimensional (1D) convolutional layer with 64 kernels, each having a window size of four and a step size of one.
2. Maxpooling layer with a window size of three and a step size of two.
3. Dropout layer with a dropout proportion of 0.2.
4. 1D convolutional layer 128 kernels, each having a window size of three and a step size of one.
5. Dropout layer with a dropout proportion of 0.2.
6. Transformer encoder with four attention heads and a 128-node forward layer.
7. Transformer encoder with four attention heads and a 128-node forward layer.
8. Transformer encoder with four attention heads and a 128-node forward layer.
9. Transformer encoder with four attention heads and a 128-node forward layer.
10. Global average layer.
11. Fully connected layer of 50 neurons with the sigmoid activation function.
12. For each cellular context, a fully connected output layer of 3 neurons with the SoftMax activation function. Eight cell contexts include DLPFC, astrocyte, endothelia, inhibitory neuron, excitatory neuron, microglia, oligodendrocyte, and oligodendrocyte progenitor cell.

The details of the model (B) are:

1. 1-dimensional (1D) convolutional layer with 962 kernels, each having a window size of four and a step size of one.
2. Maxpooling layer with a window size of three and a step size of two.
3. Dropout layer with a dropout proportion of 0.2.
4. 1D convolutional layer 360 kernels, each having a window size of three and a step size of one.
5. Dropout layer with a dropout proportion of 0.2.
6. Fully connected layer of 100 neurons with the sigmoid activation function.
7. Fully connected layer of 50 neurons with the sigmoid activation function.
8. For each cellular context, a fully connected output layer of 3 neurons with the SoftMax activation function. Eight cell contexts include DLPFC, astrocyte, endothelia, inhibitory neuron, excitatory neuron, microglia, oligodendrocyte, and oligodendrocyte progenitor cell.

The details of the model (C) are:

1. 1-dimensional (1D) convolutional layer with 64 kernels, each having a window size of four and a step size of one.
2. Maxpooling layer with a window size of three and a step size of two.
3. Dropout layer with a dropout proportion of 0.2.
4. 1D convolutional layer 128 kernels, each having a window size of three and a step size of one.
5. Dropout layer with a dropout proportion of 0.2.
6. Fully connected layer of 50 neurons with the sigmoid activation function.
7. Fully connected layer of 25 neurons with the sigmoid activation function.
8. For each cellular context, a fully connected output layer of 3 neurons with the SoftMax activation function. Eight cell contexts include DLPFC, astrocyte, endothelia, inhibitory neuron, excitatory neuron, microglia, oligodendrocyte, and oligodendrocyte progenitor cell.

We used the Rectified Linear Unit (ReLU) activation function in the convolutional and transformer layers. In the convolutional, transformer, fully connected layers, the penalty coefficients of L1 and L2 regularizations were 10^{-8} and 5×10^{-8}, respectively, and the max weight constraint of the parameters in a kernel or neuron was 0.9.

Differentially expressed genes in healthy DLPFCs.

We downloaded the RNA-seq data of 251 biosamples from the ENCODE project and 20 elderly undemented cases (the average age at death was 89 years) from the ROSMAP project (*1*). The gene expression, measured as the Reads Per Kilobase of the transcript, per Million mapped reads (RPKM values). The expression difference of a given gene between healthy DLPFC and ENCODE biosamples was measured as

$$\log_2 \frac{\text{average expression among healthy DLPFC samples}}{\text{average expression among ENCODE samples}}.$$

These values are displayed in Figs. 2A and 2B.

Allele specific analysis of CTCF ChIP-seq data.

We downloaded the CTCF ChIP-seq bam files for 61 DLPFCs from the ROSMAP project (*1*)(https://www.encodeproject.org/brain-matrix/?type=Experiment&status=released&internal_tags=RushAD). We used the samtools to read the genotypes and the numbers of allele-specific ChIP-seq reads in these samples, with the commands of "samtools merge" and "bcftools mpileup".

Tissue-specificity of genes based on gene expression profiles.

We downloaded the RNA-seq data of 251 biosamples from the ENCODE project. The gene expression, measured as the Reads Per Kilobase of the transcript, per Million mapped reads (RPKM values), was normalized so that the expression level of each gene had a median of zero. Tissue-specificity of a gene was measured using tau (τ) (*2*) as

$$\tau = \frac{\sum_{i=1}^{N}(1-\hat{x}_i)}{N}, \hat{x}_i = \frac{x_i}{max_{k=1}^{N} x_k}, \qquad (1)$$

where x_i is the expression of a tested gene in the cell line i. N is the number of the cell lines under consideration. The values of τ are in the range of $[0,1]$. A high value of τ corresponds to a large variation in gene expression across tissues, i.e., a high tissue specificity. The genes with $\tau > 0.97$ were considered as tissue-specific, while the genes with $\tau < 0.8$ were labelled as housekeeping.

TF motif analysis.

TF binding motifs were retrieved from the MEME Suite (https://meme-suite.org/meme/db/motifs). The Find Individual Motif Occurrence (FIMO), with the default setting, was used to map motifs in genomic sequences with the default setting.

The regulatory effect of a binding motif was quantified by comparing its overlap with H3K27ac ChIP-seq peaks in the healthy DLPFC to the overlap with H3K27me3 peaks. That is, for a motif (say, *m*), we have

$$\text{effect(m)} = \log_2 \frac{n_{27ac,m}/n_{27ac}}{n_{27me3,m}/n_{27me3}}. \qquad (2)$$

Here, n_{27ac} represents the numbers of adSNVs carrying H3K27ac ChIP-seq signals, while $n_{27ac,m}$ is the count of these H3K27ac adSNVs overlapping with the motif *m*. Given that H3K27ac and H3K27me3 modifications are associated with transcriptional activation and suppression, a positive/negative effect(m) denotes an activating/repressive binding motif.

Motif gain caused by a set of variants was evaluated using two-sided binomial tests, with all distal-RE adSNVs serving as control. For a given motif, we applied the Python package scipy scipy.stats.binomtest(*g*, *n*, F) to evaluate the enrichment of gain. *n* represents the number of motifs mapped to the sequences carrying risk-associated alleles, and *g* represents the number of gained motifs due to AD-associated mutations among these mapped motifs. F denotes the expected fraction of gained motifs among control variants. Similarly, the enrichment of motif

losses was evaluated using scipy.stats.binomtest(l, m, F) where m represents the number of motifs mapped to the sequences carrying protective alleles and l represents the number of lost motifs among AD-risk alleles.

Supplementary Figures

Figure S1

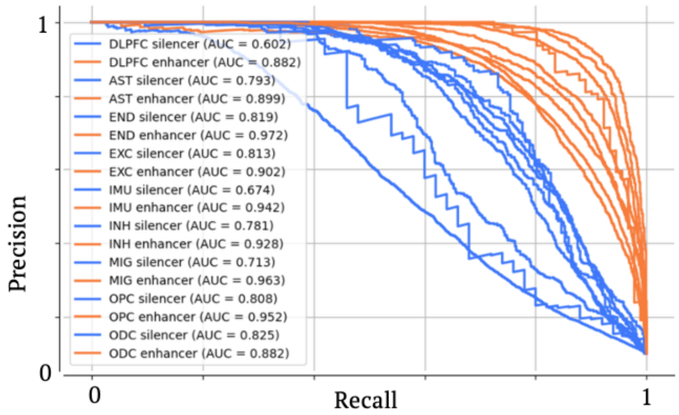

Fig. S1. Classification performance (auPRCs) of the DLPFC TREDNet for silencers and enhancers in the DLPFC and its cell types.

Figure S2

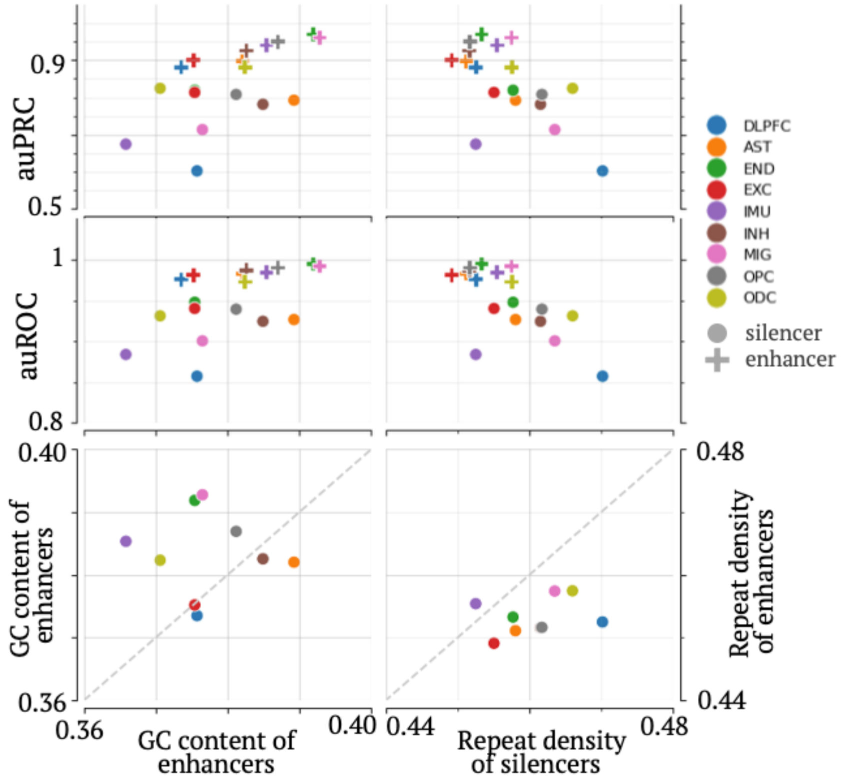

Fig. S2. Classification performance (auROC and auPRC) of the DLPFC TREDNet across brain cell types. These plots indicate the positive correlation of classification performance with GC content and negative with repeat density; and the lower GC content and higher repeat density of silencers than enhancers in DLPFC and brain cell types.

Figure S3

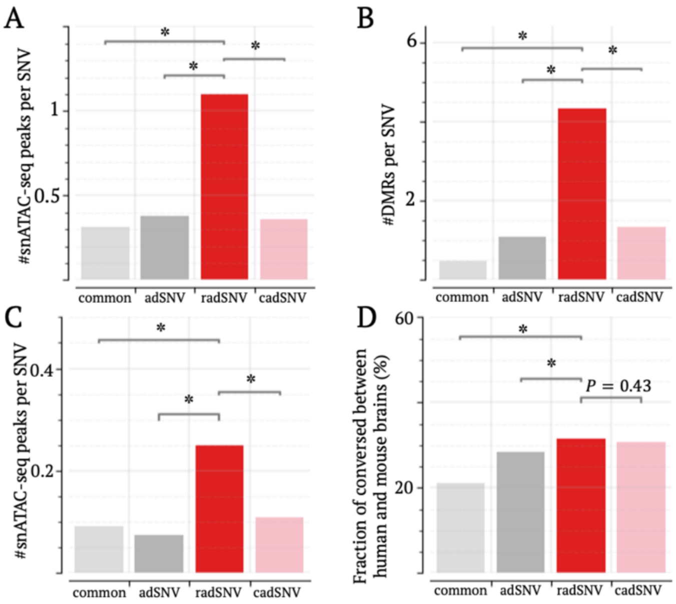

Fig. S3. Functional evaluation of radSNVs in terms of densities (A) snATAC-seq peaks in the middle temporal gyrus (*3*); (B) demethylated regions in the brain (*4*); (C) snATAC-seq peaks across brain regions (*5*); (D) snATAC-seq peaks conserved between human and mouse brains. $*P < 10^{-10}$ were determined against common SNVs using two-sided binomial tests without adjustment.

Figure S4

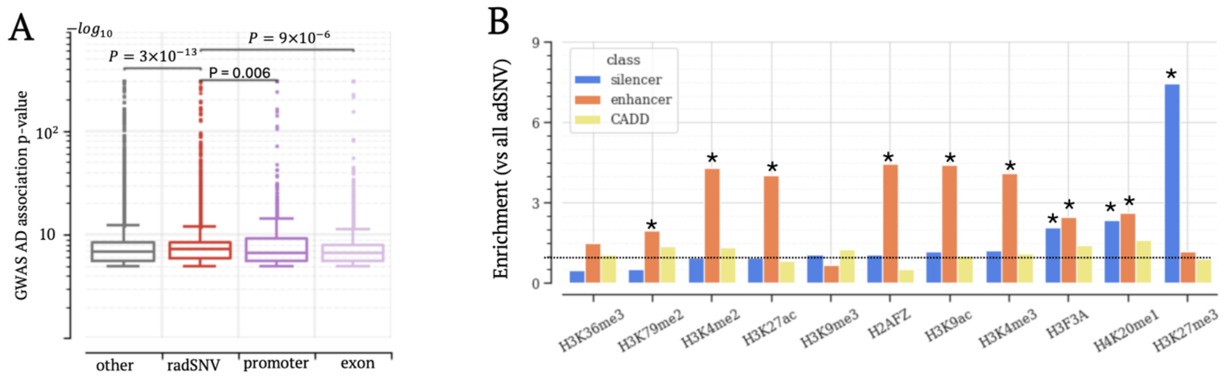

Fig. S4. Evaluation of radSNVs. (A) GWAS association significance with AD ($-\log_{10} P$) across adSNVs groups. The AD-association P value of a variant is the smallest association P-value across the examined three AD GWAS studies. P values were determined using two-sided Wilcoxon rank sum tests without adjustment. The center line in a box shows the median; the box bounds represent the lower and upper quartiles; the whiskers extend to the minima and maxima point up to a maximum of 1.5× the interquartile range. (B) Enrichment of radSNVs in histone mark ChIP-seq peaks derived in H1-differentiated neural cells. $*P < 0.0001$ were determined against all adSNVs using two-sided binomial tests without adjustment. “CADD” represents cadSNVs, while “silencer” and “enhancer” represent silencer and enhancer radSNVs, respectively.

Figure S5

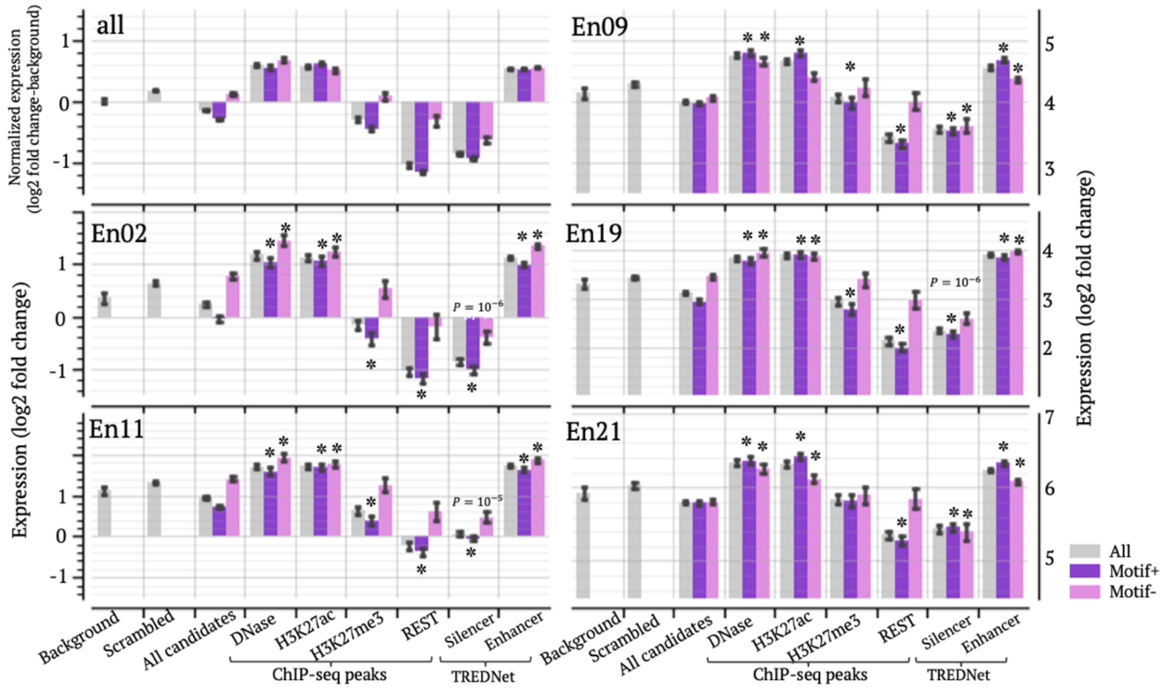

Fig. S5. Performance of the DLPFC TREDNet model in MPRAduo sequences. The top left panel summarizes the results across different constructs (the same results are presented in Figure 1H). Others show results for individual constructs, each embedded with an enhancer indicated in the left top corner. Data are presented in mean±SEM. * $P < 10^{-5}$ were determined against background (i.e, random sequences) using two-sided Wilcoxon rank sum tests without adjustment.

Figure S6

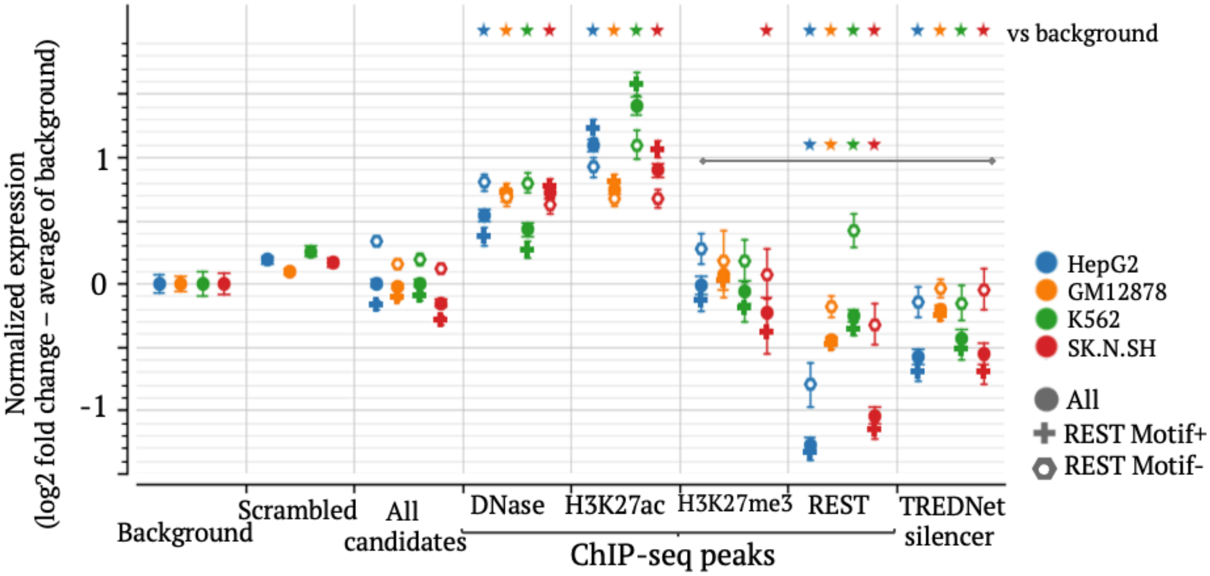

Fig. S6. Performance of the TREDNet models built for different cell types. These models were published in our previous study (*6*). Data are presented in mean ± SEM. * $P < 10^{-10}$ were determined either against background (i.e., random sequences), or between RESTmotif- and RESTmotif+ elements, using two-sided Wilcoxon rank sum tests without adjustment.

Figure S7

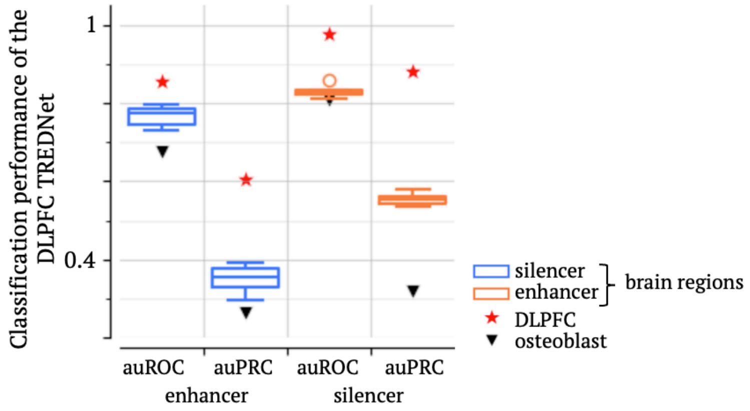

Fig. S7. Performance of the DLPFC TREDNet in other brain regions and osteoblasts. The center line in a box shows the median; the box bounds represent the lower and upper quartiles; the whiskers extend to the minima and maxima point up to a maximum of 1.5× the interquartile range.

Figure S8

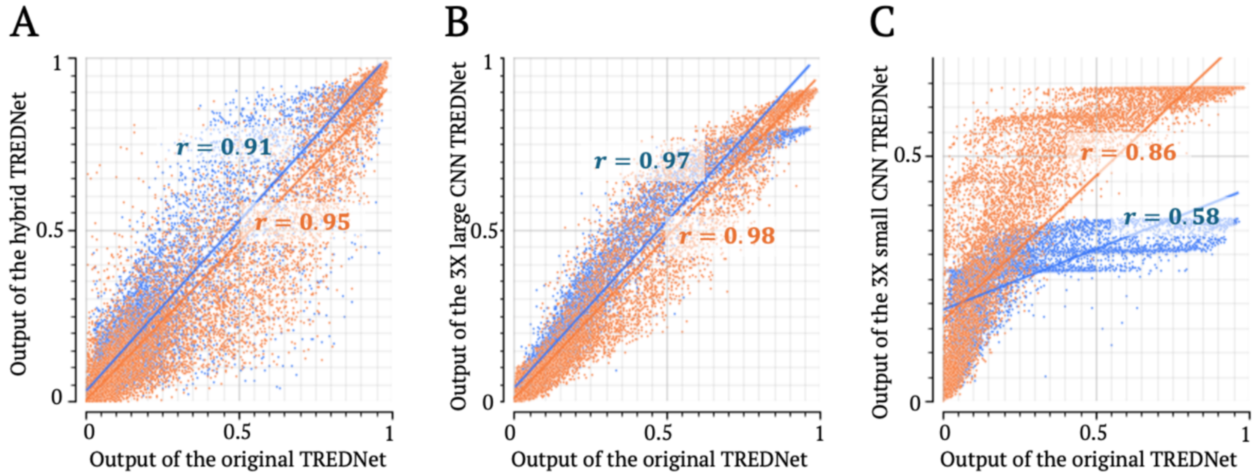

Fig. S8. Correlations of outputs between the DLPFC TREDNet and other networks (A) the hybrid model of CNN and transformer Performance; (B) the CNN with 3X more parameters and (C) the CNN with 3X fewer parameters.

Figure S9

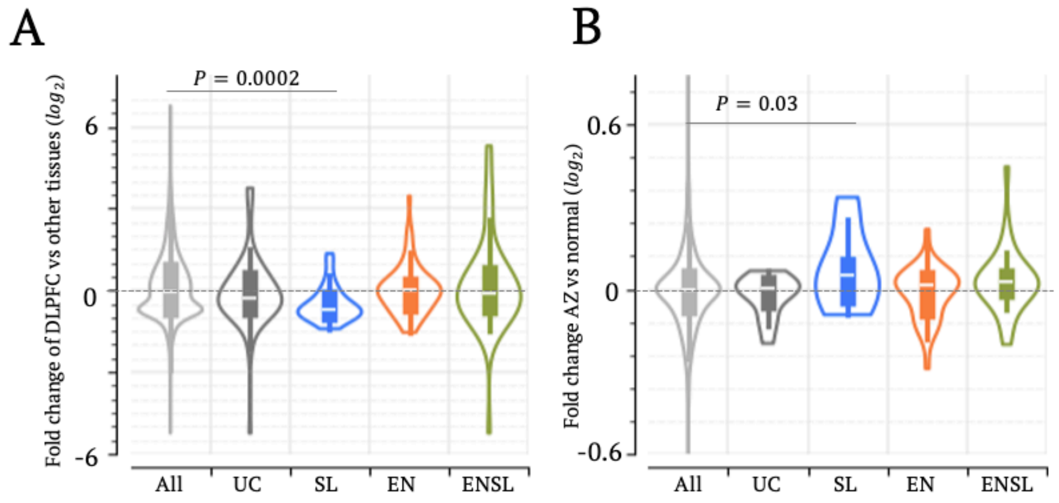

Fig. S9. Expression fold changes between healthy (**A**) and AD DLPFCs (**B**) for genes linked with different AD locus classes by Hi-C contacts. *P* values were determined against “All” (i.e., all genes with Hi-C contacts) using two-sided Wilcoxon rank sum tests without adjustment. The center line in a box shows the median; the box bounds represent the lower and upper quartiles; the whiskers extend to the minima and maxima point up to a maximum of 1.5× the interquartile range.

Figure S10

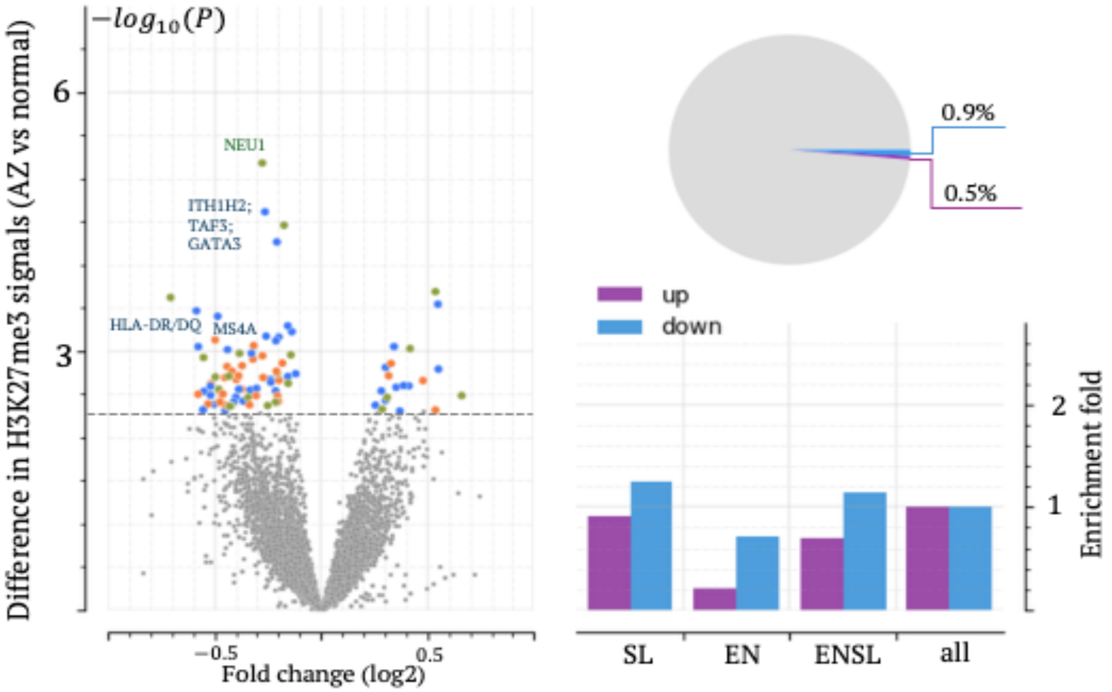

Fig. S10. Changes in H3K27me3 signals between healthy and AD DLPFCs (the volcano plot in the left panel), and their distribution across different locus classes (the bar plot and the pie chart in the right panel). In the volcano plot, each dot represents a H3K27me3 peak. Grey dots indicate a peak with an insignificant change. Blue, orange, and blue dots indicate peaks with significant changes located in SL, EN, and ENSL loci, respectively.

Figure S11

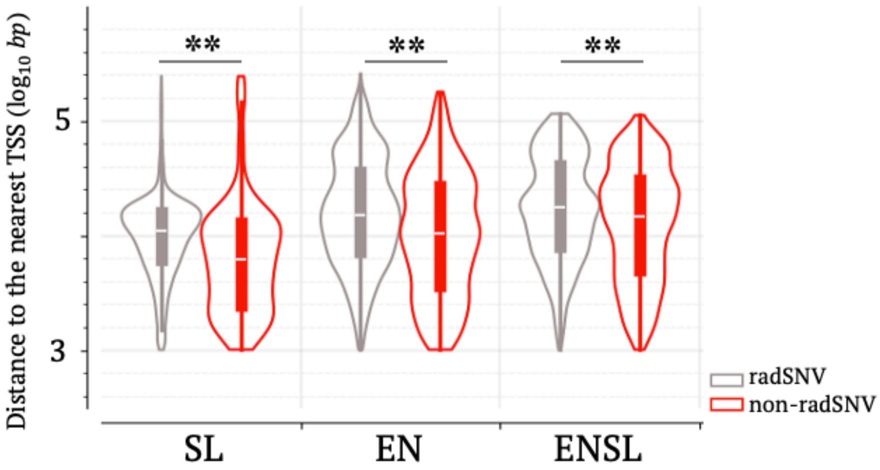

Fig. S11. Distances of radSNVs to their nearest TSSs (in bp). Non-radSNV presented distal-RE adSNVs that are located in H3K27ac or H3K27me3 peaks but not predicted as radSNVs. $** P < 10^{-10}$ were determined using two-sided Wilcoxon rank sum tests without adjustment. The center line in a box shows the median; the box bounds represent the lower and upper quartiles; the whiskers extend to the minima and maxima point up to a maximum of 1.5× the interquartile range.

Figure S12

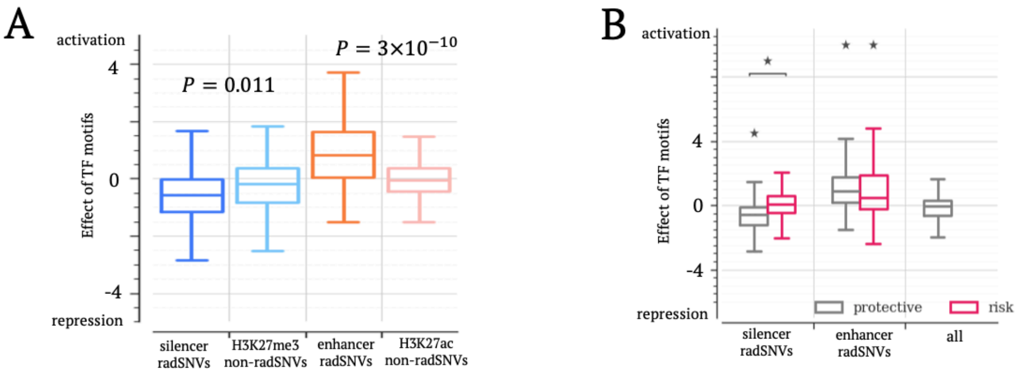

Fig. S12. Effect scores of TF motifs enriched among adSNV sets. (A) Motifs enriched in silencer and enhancer radSNVs with protective alleles. Presented P values are the significant difference between radSNVs and their non-radSNV counterparts. Non-radSNVs are the adSNVs located in histone mark peaks but not predicted as radSNVs. *P* values were determined using two-sided Wilcoxon rank sum tests without adjustment. The center line in a box shows the median; the box bounds represent the lower and upper quartiles; the whiskers extend to the minima and maxima point up to a maximum of 1.5× the interquartile range. **(**B) Motifs enriched in silencer and enhancer radSVNs with protective or risk alleles. Controls for motif enrichment analysis are all distal-RE adSNVs (denoted as "all" in the plot). *P* values ($*P < 10^{-5}$) were determined against all (if not specified) using two-sided Wilcoxon rank sum tests without adjustment. In this figure, the center line in a box shows the median; the box bounds represent the lower and upper quartiles; the whiskers extend to the minima and maxima points up to a maximum of 1.5× the interquartile range.

Figure S13

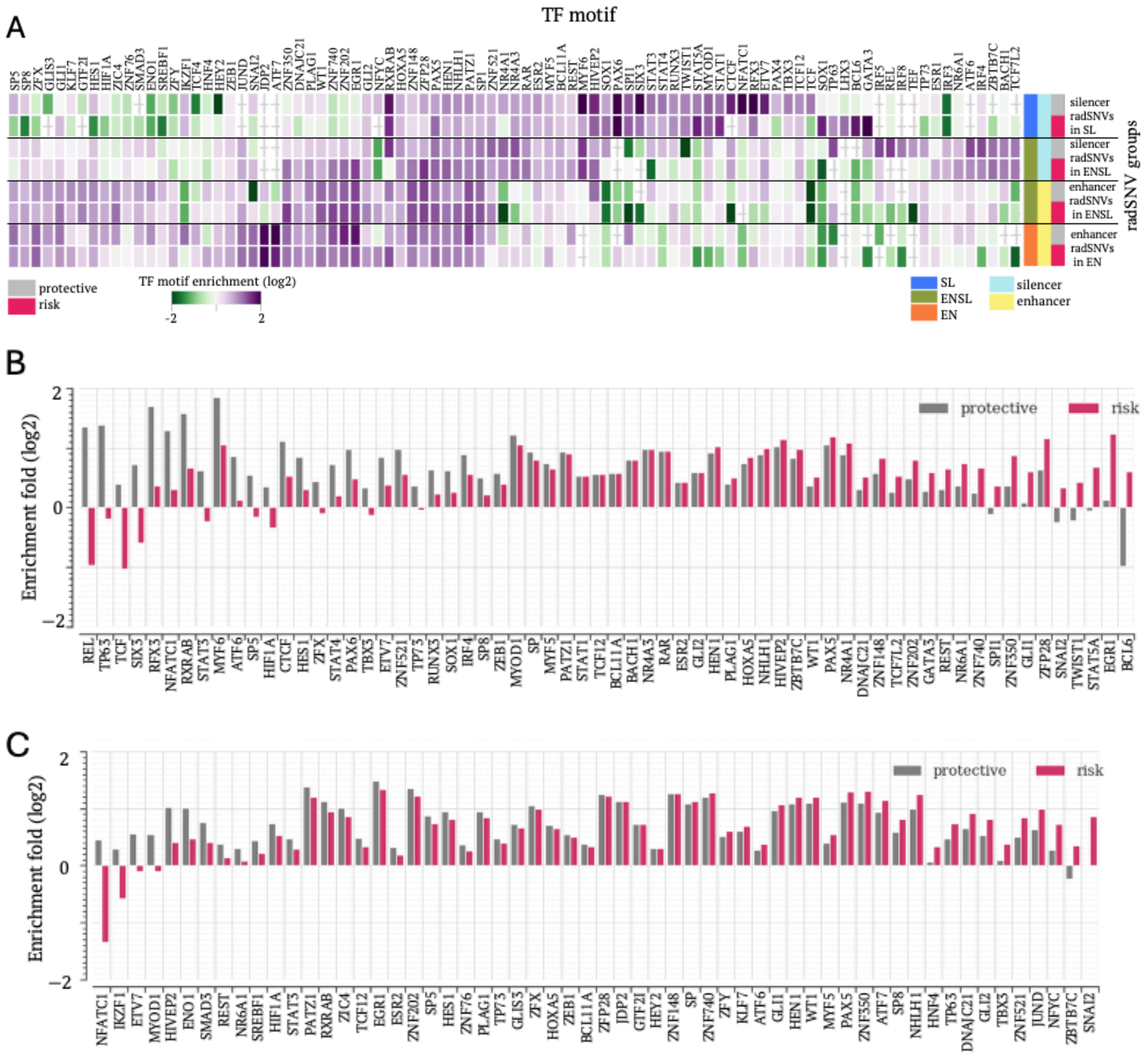

Fig. S13. TF motifs enriched in radSNVs with protective and risk alleles. (A) Motif enrichments across radSNV classes. Motif enrichment comparisons between protective and risk alleles among (B) silencer and (C) enhancer radSNVs. Controls for motif enrichment analysis are all regulatory adSNVs.

Figure S14

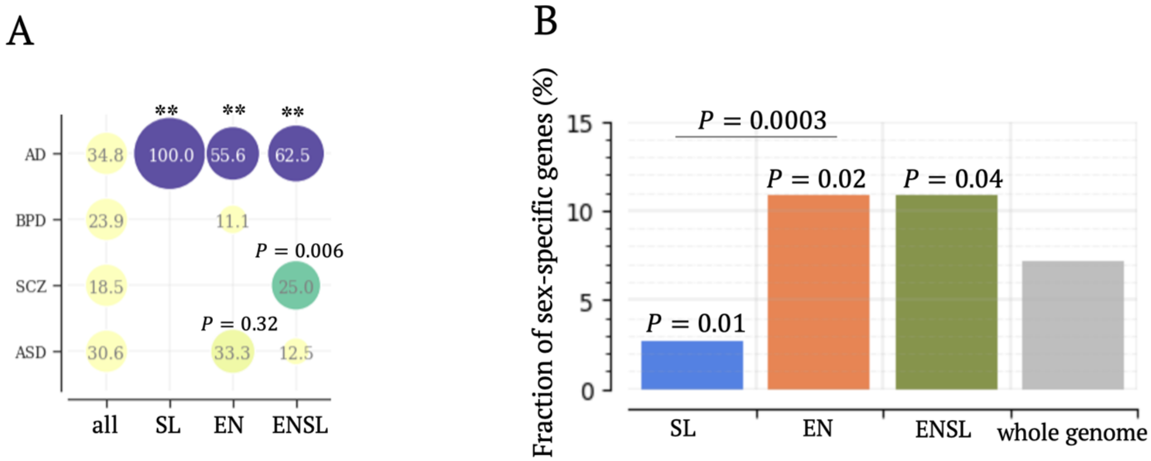

Fig. S14. (A) Enrichment of disease-associated genes in AD susceptibility locus classes. *P* values ($** P < 10^{-10}$) were determined against all adSNVs using two-sided binomial tests without adjustment. (B) Fraction of sex-specific AD-associated genes across locus classes. P-values above bars indicate the significance as compared to the whole genome.

Figure S15

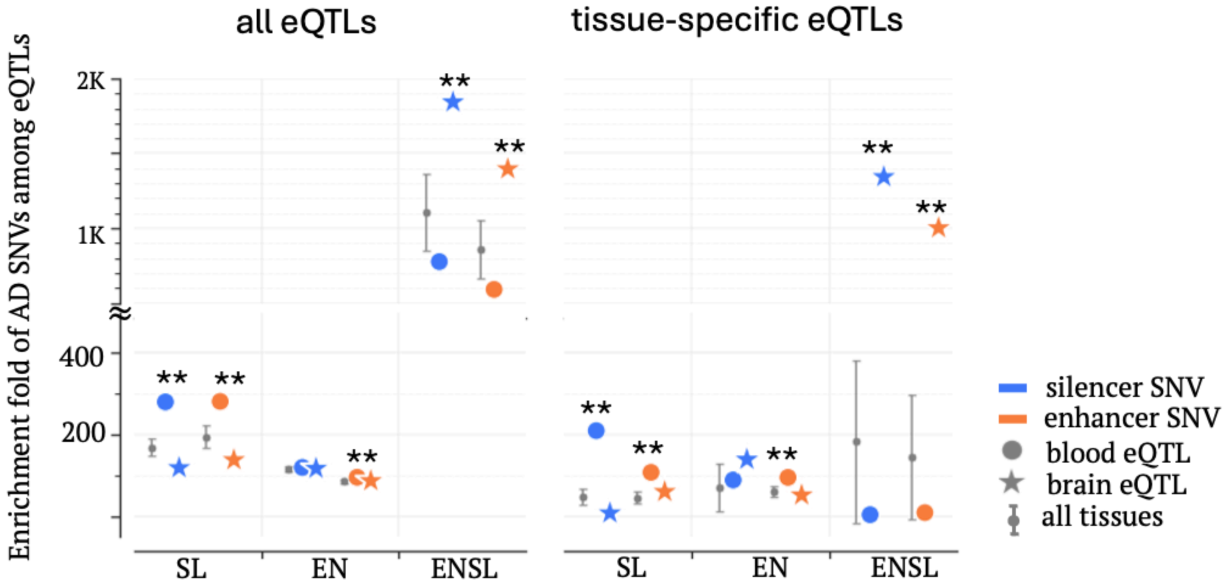

Fig. S15. Enrichment of radSNVs among eQTLs in brain and blood across locus classes. RadSNVs of a locus class were stratified into two groups: silencer SNVs and enhancer SNVs. The results demonstrate the comparable regulatory importance between silencer and enhancer SNVs. Data are presented with the median±SEM. *P* values were determined against all tissues (n=22) using the two-sided Student's *t*-tests without adjustment.

Figure S16

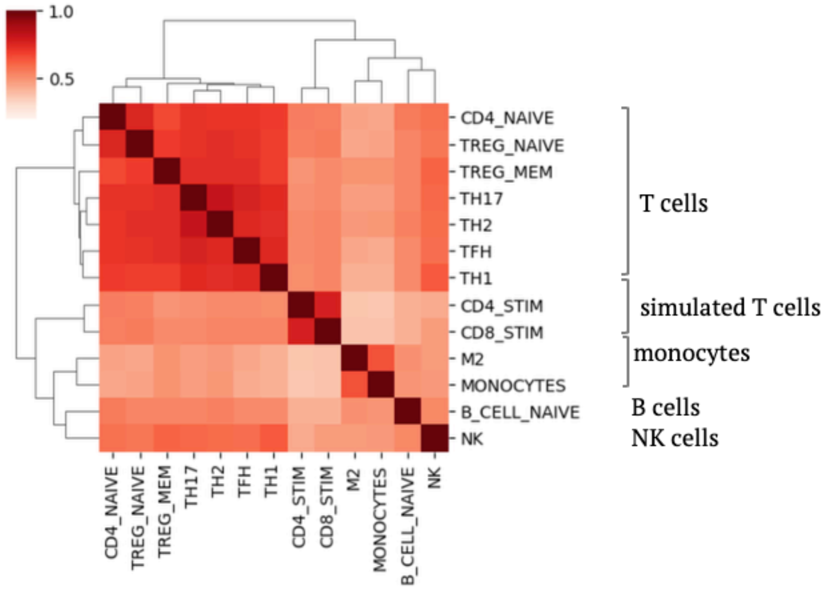

Fig. S16. Similarities of DICE eQTLs across blood cell types. Based on these similarities, these blood cell types are clustered in five groups. Jaccard index was used to measure the similarities (i.e., the overlaps) between eQTL sets of blood cell types.

Figure S17

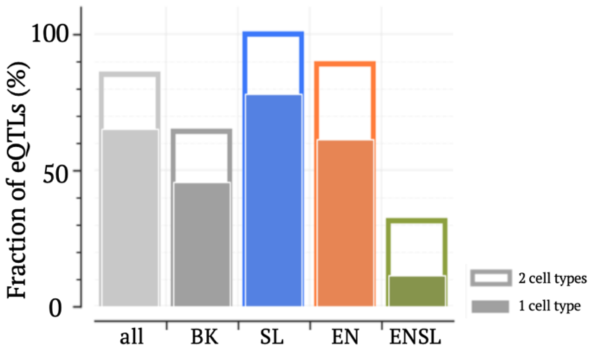

Fig. S17. Cell-specificity of brain eQTLs across locus classes. Cell-specificity of an eQTL is measured as the number of cell types where that eQTL is active.

Figure S18

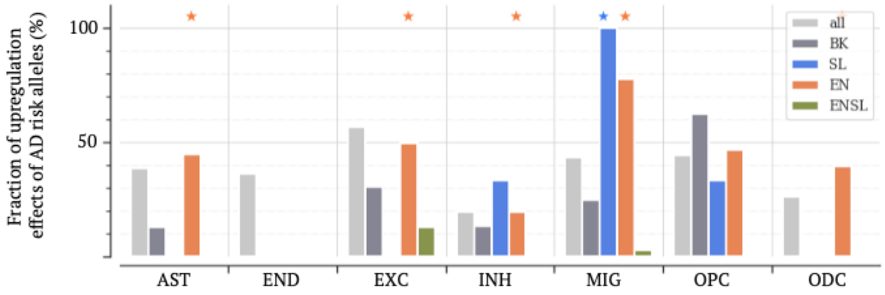

Fig. S18. Proportions of eQTL radSNVs where AD risk alleles are associated with increased gene expression. P values ($* \, P < 10^{-10}$) were determined against all adSNVs using two-sided binomial tests without adjustment.

Figure S19

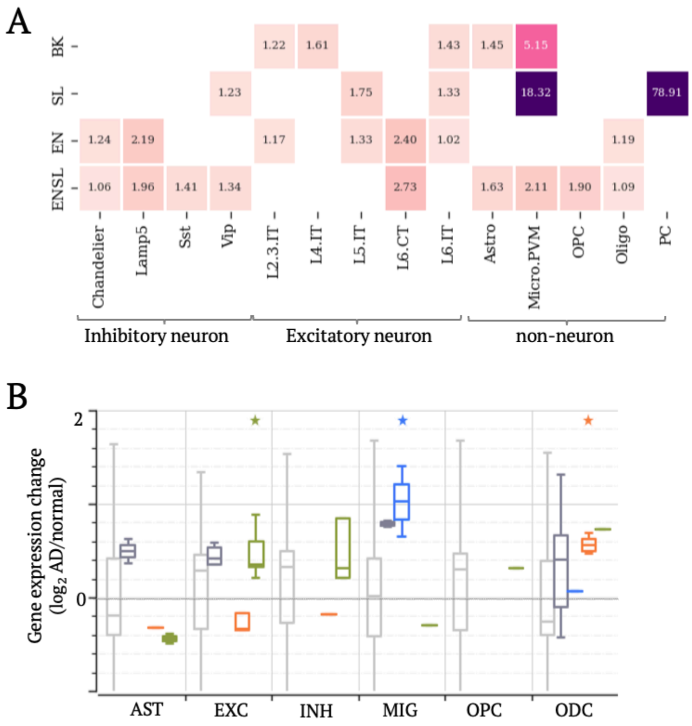

scQTL cell type	scRNA-seq cell type
Chandelier	Inhibitory neuron
Lamp5	
Sst	
Vip	
L2.3.IT	Excitatory neuron
L4.IT	
L5.IT	
L6.CT	
L6.IT	
Astro	Astro
Micro.PVM	Microglia
PC	
OPC	OPC
Oligo	Oligo

Fig. S19. Results based on the brain single-cell eQTL dataset reported by Emani P. S. et al. (*7*) (A) Enrichment of radSNVs among scQTLs across locus classes. (B) Expression changes in AD brains of eQTL genes associated with radSNVs in different locus classes across brain cell types. We used the table next to (B) to map cell types examined in eQTLs to those in AD scRNA-seq data. P values ($* \, P < 10^{-5}$) were determined against all examined genes using two-sided Wilcoxon rank sum tests without adjustment. The center line in a box shows the median; the box bounds represent the lower and upper quartiles; the whiskers extend to the minima and maxima points up to a maximum of 1.5× the interquartile range.

Figure S20

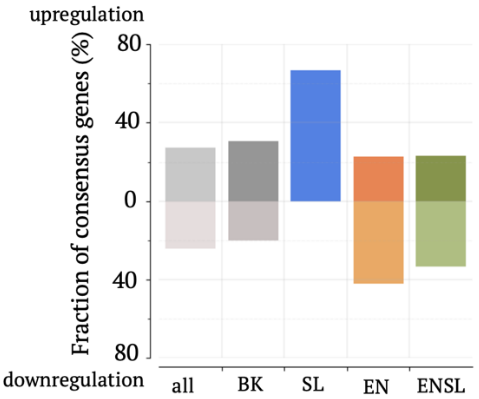

Fig. S20. Fraction of INFβ-up/down-regulated genes that are induced/repressed by pre-formed Aβ fibrils across locus classes.

Figure S21

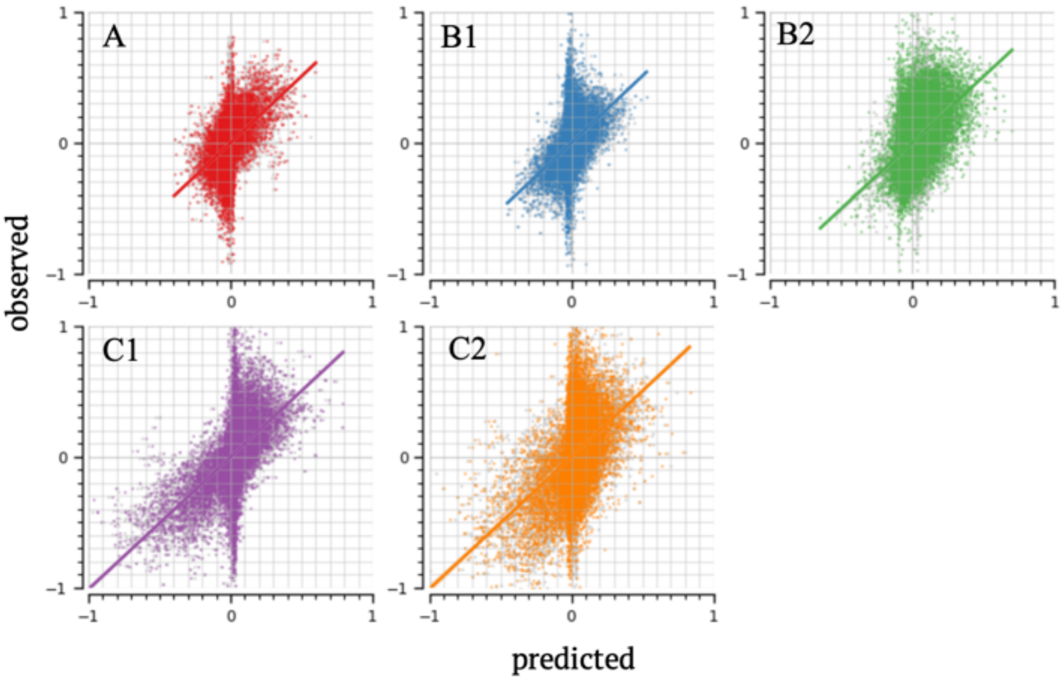

Fig. S21. Comparison of observed and predicted gene expression across AD subtypes.

Figure S22

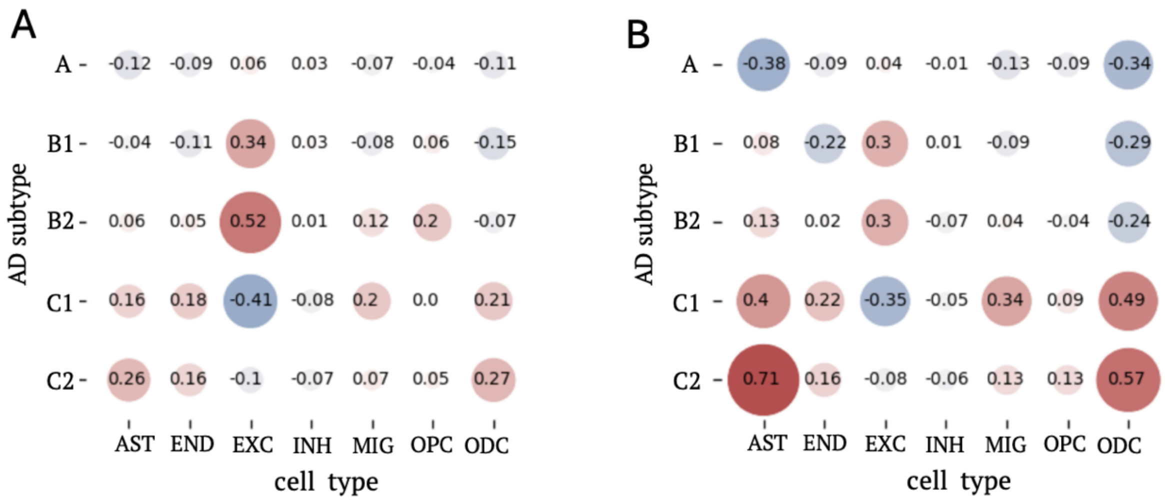

Fig. S22. Average weights in linear regression models built on (A) cell-specific gene expression a and (B) enhancer/silencer profiles.

Figure S23

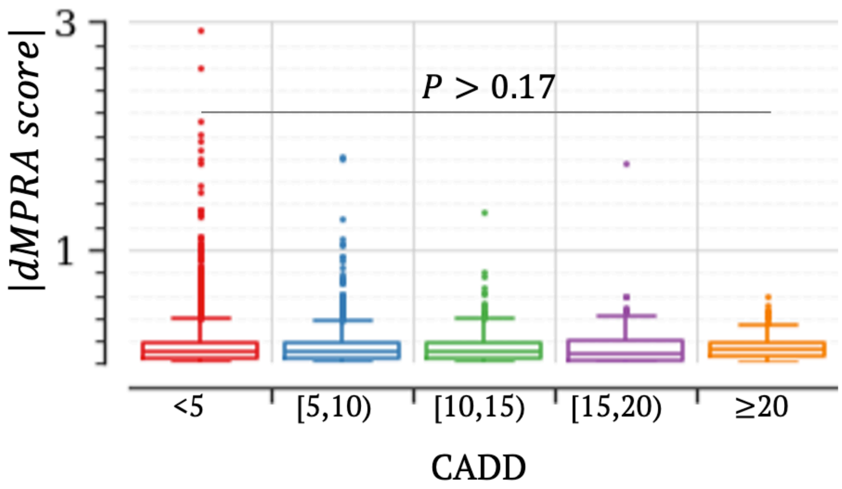

Fig. S23. Distribution of $|dMPRA\ score|$ for different CADD ranges. P values were determined using two-sided Wilcoxon rank sum tests without adjustment. The center line in a box shows the median; the box bounds represent the lower and upper quartiles; the whiskers extend to the minima and maxima point up to a maximum of 1.5× the interquartile range.

Figure S24

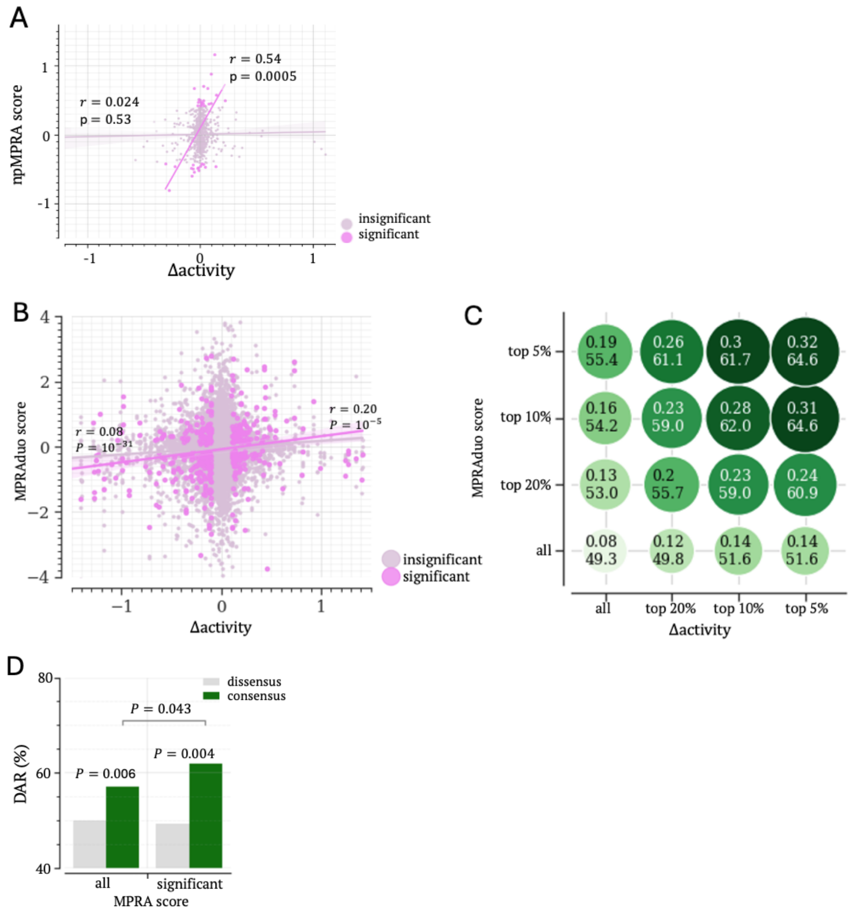

Fig. S24. Correlation between $\Delta activity$ scores and MPRA results derived in two other studies: (A) npMPA (*8*) and (B) MPRAduo (*9*). (C) DCRs between $\Delta activity$ and MPRAduo scores for different cutoff settings. (D) DCRs for variants tested in multiple MPRA studies. *P* values were determined using two-sided binomial tests without adjustment.

Figure S25

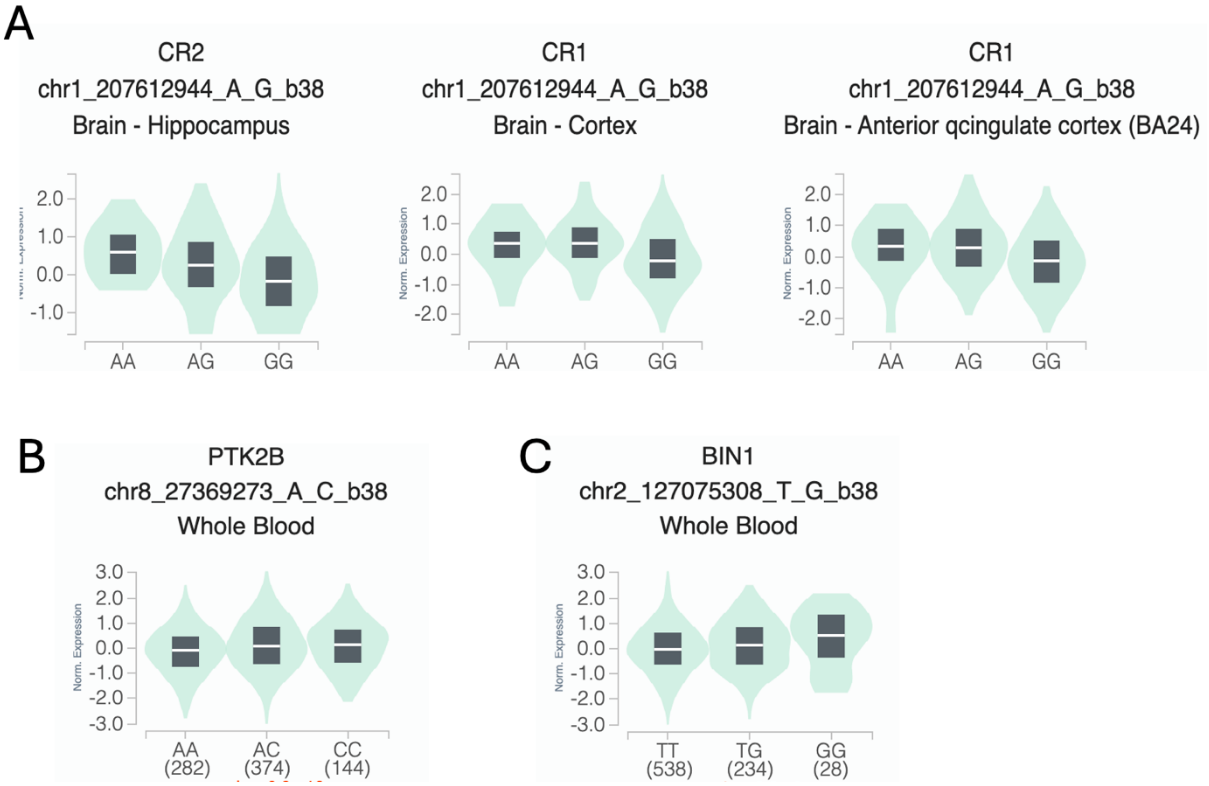

Fig. S25. GTEx results for eQTLs (A) rs6701713 (B) rs755951 and (C) rs6705877.

Figure S26

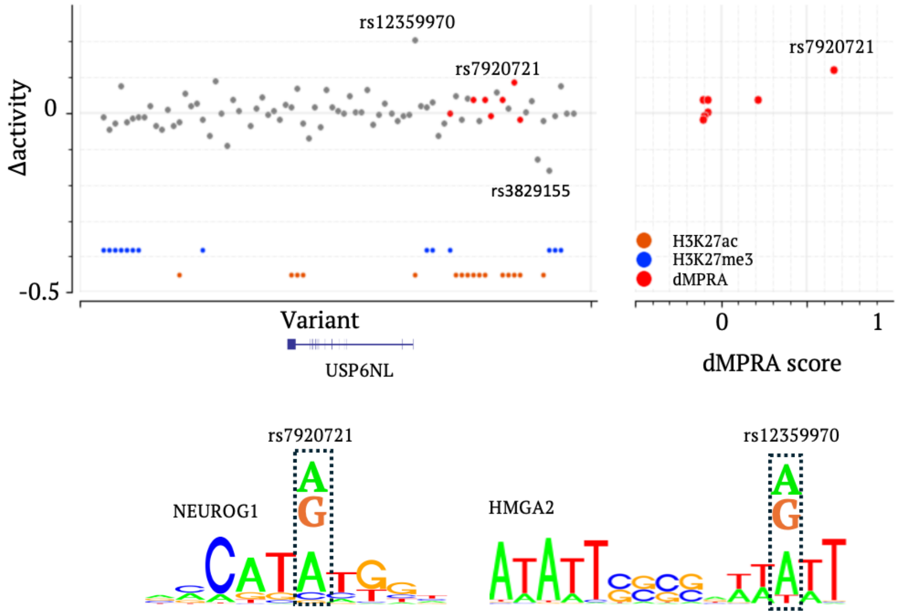

Fig. S26. Prioritizing radSNVs in the *USP6NL* locus.

Table S1. List of AD susceptibility loci. (separate file)

Table S2. auROCs and auPRCs of the DLPFC TREDNet on testing samples. (separate file)

Table S3. Annotations of all adSNVs. Predicted $\Delta activity$ scores of these variants, together with MPRA scores, Hi-C target genes, allele-specific motif mappings, are provided. (separate file)

Table S4. H3K27ac and H3K27me3 peaks having significantly different intensities between healthy and AD cases. (separate file)

Table S5. Motif gain and loss enrichments among putative causal radSNVs. (separate file)

Table S6. Prediction results on CRISPR-validated MPRA SNVs. (separate file)
